# Hard-to-reach populations of men who have sex with men and sex workers: a systematic review on sampling methods

**DOI:** 10.1186/s13643-015-0129-9

**Published:** 2015-10-30

**Authors:** Ana B. Barros, Sonia F. Dias, Maria Rosario O. Martins

**Affiliations:** Lúrio University, Rua 4250, Km 2.3, Marrere, Nampula Mozambique; Global Health and Tropical Medicine, Instituto de Higiene e Medicina Tropical, Universidade Nova de Lisboa, Rua da Junqueira 100, Lisbon, Portugal

**Keywords:** Hard to reach populations, Men who have sex with men, Sex workers, Sampling methods

## Abstract

**Background:**

In public health, hard-to-reach populations are often recruited by non-probabilistic sampling methods that produce biased results. In order to overcome this, several sampling methods have been improved and developed in the last years. The aim of this systematic review was to identify all current methods used to survey most-at-risk populations of men who have sex with men and sex workers. The review also aimed to assess if there were any relations between the study populations and the sampling methods used to recruit them. Lastly, we wanted to assess if the number of publications originated in middle and low human development (MLHD) countries had been increasing in the last years.

**Methods:**

A systematic review was conducted using electronic databases and a total of 268 published studies were included in the analysis.

**Results:**

In this review, 11 recruitment methods were identified. Semi-probabilistic methods were used most commonly to survey men who have sex with men, and the use of the Internet was the method that gathered more respondents. We found that female sex workers were more frequently recruited through non-probabilistic methods than men who have sex with men (odds = 2.2; *p* < 0.05; confidence interval (CI) [1.1–4.2]). In the last 6 years, the number of studies based in middle and low human development countries increased more than the number of studies based in very high and high human development countries (odds = 2.5; *p* < 0.05; CI [1.3–4.9]).

**Conclusions:**

This systematic literature review identified 11 methods used to sample men who have sex with men and female sex workers. There is an association between the type of sampling method and the population being studied. The number of studies based in middle and low human development countries has increased in the last 6 years of this study.

**Electronic supplementary material:**

The online version of this article (doi:10.1186/s13643-015-0129-9) contains supplementary material, which is available to authorized users.

## Background

In public health, hard-to-reach populations (HRP), hidden populations [1] or most-at-risk populations [2] are mainly associated with illegal or stigmatizing behaviours such as sex workers (SW), injection drug users (IDU), men who have sex with men (MSM) or homeless people [3, 4]. These groups are usually seen as key populations to be targeted as they have an important role on the spread of communicable diseases like HIV or tuberculosis [5–7]. Thus, understanding how infectious epidemics affect them is crucial for the development of targeted and successful public health interventions. Ideally, a representative sample of the study population should be selected and their socio-demographic characteristics and risk behaviours identified. However, most HRP do not have a sampling frame because their members are “hidden”; hence, one cannot count how many they are [8, 9]. On the other hand, population-based surveys need to be very large to include enough “hidden” members to get precise estimates, which is a limiting factor mainly due to high costs. Therefore, studying HRP presents several difficulties and challenges: (a) it is extremely difficult to use probability sampling strategies to choose members to be included in the sample, and consequently, non-probabilistic sampling methods are mainly used. The great disadvantage of these methods is that since they do not select individuals randomly, the chosen elements may not be representative of the population to which they belong [10]; (b) each HRP has its own behavioural characteristics and deciding on which methods are the most adequate to use in each population is not straightforward [2]; (c) in spite of the financial support most middle and low human development (MLHD) countries have been receiving for infectious diseases control [11], they still receive inadequate funding to reduce the vulnerability of HRP [12]. Consequently, the need persists for documenting trends on the HIV epidemics for these key populations in these regions [13, 14].

Although several studies have been done in reviewing sampling methods [10, 15–18], we did not find any systematic literature review.

The aim of this systematic review was to identify all current methods used to survey the most-at-risk populations of MSM and SW. The review also aimed to assess if there was any relation between the study populations and the sampling methods used to recruit them (that is, to find if there is statistical significance between study populations and sampling methods). Lastly, we wanted to assess if the number of publications originated in MLHD countries had been increasing in the last years.

### Data definitions

#### Populations

In public health, MSM is a term used to define men who engage in sex with other men irrespective of their sexual and gender identities. Commonly, this definition includes men who are identifies as gay, homosexual, bisexual, heterosexual and transgendered [19].

A transgender person (TG) is someone who has a gender identity different from his/her sex at birth. Transgender people may be male to female (female appearance) or female to male (male appearance) [20]. In this systematic review, the term “transgender” refers to the former definition because we did not find any study related to the latter.

Sex worker (SW) is defined as a person who receives money or goods in exchange for sexual services and encompasses male (MSW), female (FSW) or transgender (TSW) people [21].

The term MSM is widely used in the literature not only to mean men who engage in sex with other men but also men sex workers (MSW) and transgender people (sex workers or not) [22]. In order to be consistent with the current literature and for the purpose of analysis, retrieved publications were divided into subgroups consisting of female sex workers and men who have sex with men. Included in this last category are studies of male sex workers, transgendered persons, transgender sex workers and men who have sex with men.

#### Sampling methods

We call recruitment methods the techniques applied to select a sample of elements from a target population. In this systematic literature review, we identified 11 recruitment methods, which we grouped into three categories. More information about the retrieved methods can be seen in Additional file 5.

The first category includes *non*-*probabilistic sampling methods* where the sampled elements are chosen arbitrarily or casually. In these methods, it is not possible to estimate the probability of each element being included in the sample, and consequently, there is no way of making inferences to the population [3]. Methods that encompass the non-probabilistic category are convenience, purposive, snowball and targeted sampling.

The second category includes *probabilistic sampling methods*. These *methods* include those where every element in the population has a known probability of being included in the sample; the concept of p*robability sampling* means that a sample has been drawn in a probabilistic way [23], and consequently, reliable estimates are produced and inferences can be made to the study population. Random digit dialling (RDD), cluster sampling, multi-stage sampling and stratified probability sampling (SPS) are included in this probabilistic category.

The third category is the *semi*-*probabilistic*; this category includes methods that we believe do not fall in either of the other two because, from a *theoretical* point of view, it is possible to determine the probability of each element being included in the sample; however, in practice, probabilities cannot be calculated [15, 24] and therefore these methods do not allow making *reliable* inferences to the (unknown) population. Internet sampling, respondent-driven sampling (RDS) and time location sampling (TLS) are included in this category.

#### Countries

Countries where the studies were conducted were first classified into eight UNAIDS regions [25]. Later, for the purpose of the analysis, these countries were classified in accordance with the development level, as defined by United Nations Procurement Division (UNPD) [26]. UNPD classifies countries in four levels of human development: very high human development (VHHD), high human development (HHD), medium human development (MHD) and low human development (LHD). For our analysis, we grouped the first two categories into one and named it as “very high and high human development (VHHHD)” and grouped the last two categories into another one and named it “medium and low human development (MLHD)”.

## Methods

The review was reported according to PRISMA recommendations [27].

### Study selection

Electronic literature searches were conducted from 1 January 2003 to 31 December 2013 in the following databases: EBSCO, Gale, NLM (PubMed Central, PMC, Medline), Oxford University Press, Ex Libris, Web of Knowledge, Elsevier, SpringerLink, Taylor & Francis Online, PLoS and SAGE. The search engine b-on—Online Knowledge Library^1^ was used to conduct database searches. The last search was run on 16 June 2014. Studies were excluded if they did not include study participants (e.g., studies that make theoretical assumptions only) and were systematic or non-systematic reviews, letters, editorials or commentaries. We also excluded clinical trials due to the very specific methods used to recruit participants, publications that did not mention the recruitment method and all publications that required additional payment for access.

In this first systematic literature review of methods used for sampling HRP, inclusion criteria were broad to cover as many publications as possible. The following list of key words was drawn up and was used as search terms: Men who have Sex with Men, Sex Work, Sex Workers, recruit, recruited, participants, enrol, enrolled, sample, sampling. The search terms Men who have Sex with Men and Sex Work and Sex Workers were included in the publication title, and in the abstract, the remaining terms were included. In this manner, papers were selected if they mentioned at least one of the two above mentioned HRP and not less than two of the remaining search terms in the abstract according to the following two steps:

- The first step was using the b-on database (capital letters indicate the search engine option): GLOBAL SEARCH; TITLE (EXACT): {sex work} OR {sex workers} OR {men who have sex with men}; ANY (CONTAINS): [{recruit} OR {recruited} OR {participants}] AND [{enrol} OR {enrolled} OR {participants}] AND [{sample} OR {sampling}]; TYPE OF MATERIAL: all publications; LANGUAGE: English; START DATE: 01/01/2003; END DATE: 31/12/2013. This search yielded 1707 publications.
- The second step was used to narrow the initial search because the b-on search engine does not have the option to search in the abstract only. Therefore, retrieved publications were exported to the Reference Manager 12© software and the same key words were applied to the abstracts (capital letters indicate the search engine option): TITLE: [{sex work} OR {sex workers} OR {men who have sex with men}] AND ABSTRACT: [{recruit} OR {recruited} OR {participants}] AND [{enrol} OR {enrolled} OR {participants}] AND [{sample} OR {sampling}]. This search identified 602 publications.

After duplicates had been deleted, titles and abstracts of all records were screened and exclusion and inclusion criteria were applied. Selected studies could use any design (other than randomized trial), must have referred to MSM and SW populations and have been public health related. Studies must have been written in English and be published between the beginning of 2003 and the end of 2013. A second level of screening was applied to the full text for those studies that did not mention the sampling method in the title and/or abstract.

We only included studies that explicitly mentioned the recruitment method. The same reasoning was applied to those studies that used more than one recruitment method but only identified one of them, for instance, only the identified method was included in the review. One reviewer initially applied the exclusion and inclusion criteria in the first and second levels of the screening, and then the second and third reviewers independently screened any studies that the first reviewer excluded to ensure that no relevant studies were accidentally left out of the review. The second and third reviewers also screened at random 20 % of the studies that were included in the first and second levels of screening to make sure the decision of inclusion was the correct one. Disagreements were resolved through discussion among all authors.

After selecting studies to be included in the systematic review, data about year of publication, country or region where the study took place, study population, sample size, and recruitment method were extracted to a spreadsheet by the first reviewer and then the second and third reviewers checked the extracted data for all studies. Disagreements were resolved through discussion among the authors.

Our study includes only descriptive data, and therefore no assessment of risk of bias was done in individual studies or across studies.

### Statistical methods

Statistical software SPSS® 22 for Mac OS was used to run the analysis. Descriptive statistics were used to analyse data. Odds ratio (OR) were calculated through logistic regression tool using the Wald chi-square test and 95 % confidence intervals (CI) were measured in order to assess if there was any association between studied populations and sampling methods and to appraise if the number of publications was associated with the regions where studies were carried out. *P* values less than 0.05 were considered statistically significant.

## Results

### Study selection

**Fig. 1:**
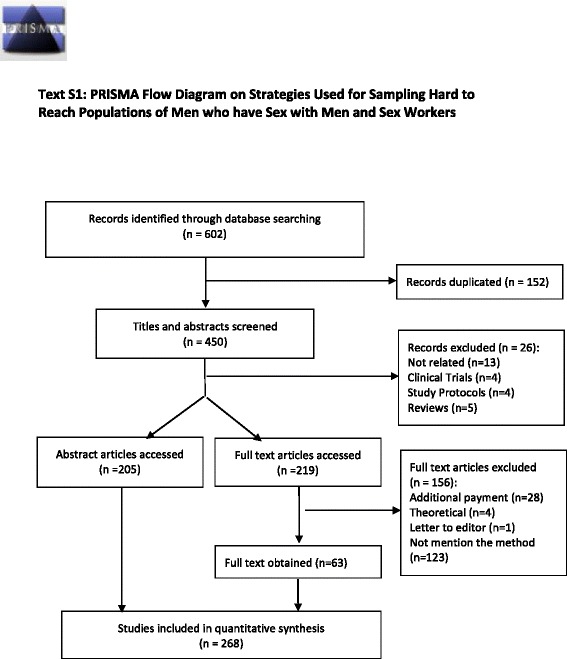
PRISMA flow diagram on strategies used for sampling hard-to-reach populations in public health

Figure 1 presents the study selection process according to PRISMA 2009 flow diagram [27]. The PRISMA checklist can be found in the Additional file 3: Text S2. Our search identified 602 records of which 152 were removed as being duplicated. After screening titles and abstracts, 26 records were excluded for the reasons stated in the flow diagram, and the recruitment methods were identified in 205 publications. During the process of screening, the full text of the remaining 219 publications and another 156 articles were excluded and 63 publications were accessed. In the end, this review included 268 published articles for the analysis.

### Study characteristics and findings

#### Recruitment methods

Table 1 presents the 11 sampling methods identified in this review. RDS method was identified in 28.7 % (*n* = 77) of publications, and TLS was mentioned in 15.7 % (*n* = 42). Snowball sampling was used as a recruitment method in 13.4 % (*n* = 36), and convenience and Internet methods were identified in 12.3 % (*n* = 33) of the publications. Targeted sampling and purposive sampling were mentioned in 5.6 (*n* = 15) and 2.2 % (*n* = 6) of retrieved publications, respectively. Multi-stage sampling, cluster sampling and RDD were identified in 1.5 (*n* = 4), 1.1 (*n* = 3) and 0.7 % (*n* = 2) of the studies, respectively. In the remaining 6.3 % of the studies, two or more methods were identified in each study and the stratified sampling was used in one of these studies.

**Table 1:** Descriptive statistics of the retrieved publications

Recruitment methods	Number of studies	Percent	Total sample sizes	Mean	Median	Minimum	Maximum	Study reference^a^
RDS	77	28.7	82,004	1065	496	50	18,960	[1–39] [40–77]
TLS	42	15.7	55,193	1346.2	526	200	16,270	[78–119]
Convenience	33	12.3	18,698	584.3	305.5	36	2569	[120–152]
Snowball	36	13.4	18,148	504.1	331.5	20	3314	[153–188]
Internet	33	12.3	225,320	6827.9	522	32	144,177	[189–221]
Targeted	15	5.6	6197	413.1	485	22	806	[222–236]
Purposive	6	2.2	195	32.5	26.5	17	58	[237–242]
Multi-stage probability sample	4	1.5	3175	793.8	216	121	2622	[243–246]
Cluster	3	1.1	1901	633.7	504	324	1073	[247–249]
Convenience, snowball	3	1.1	687	229	253	161	273	[250–252]
TLS, Internet	2	0.7	1167	583.5	583.5	566	601	[253, 254]
RDD	2	0.7	2659	1329.5	1329.5	879	1780	[255, 256]
Convenience, Internet	2	0.7	2291	1145.5	1145.5	770	1521	[257, 258]
Convenience, RDD	1	0.4	218					[259]
Convenience, RDS	1	0.4	624					[260]
Internet, snowball	1	0.4	1692					[261]
RDS, TLS	2	0.7	1853	926.5	926.5	737	1116	[262, 263]
Stratified probability sampling, Internet	1	0.4	2182					[264]
Targeted, snowball	1	0.4	48					[265]
Convenience, snowball, Internet	1	0.4	103					[266]
Snowball, TLS, RDS	1	0.4	1407					[267]
RDS, Internet	1	0.4	2147					[268]
Total	268	100	427,909					

^a^References can be found in Additional file 4: Text S3

Internet was the sampling method that gathered more respondents with a total of 225,320 participants in 33 selected studies. RDS gathered 82,004 respondents in 77 studies and 55,193 respondents participated in the 42 studies that used the TLS method. Altogether, these three sampling methods recruited more than 84 % of all respondents identified in the publications.

The identified recruitment methods were categorized into one of three categories (Table 2): non-probabilistic (*n* = 106), *semi*-*probabilistic* (*n* = 170) and probabilistic (*n* = 11). Methods included in the *semi*-*probabilistic category*, Internet, TLS and RDS were applied in the most retrieved studies (59 %) and were also those who recruited more respondents (more than 80 %).

**Table 2:** Recruitment methods by categories

Recruitment methods	Form	Category
Convenience	Venue-based	Non-probabilistic
Purposive	Link-tracing	Non-probabilistic
Snowball	Link-tracing	Non-probabilistic
Targeted	Link-tracing	Non-probabilistic
Internet	Venue-based	Semi-probabilistic
RDS	Link-tracing	Semi-probabilistic
TLS	Venue-based	Semi-probabilistic
Cluster	Probabilistic	Probabilistic
Multi-stage probability sample	Probabilistic	Probabilistic
RDD	Probabilistic	Probabilistic
Stratified probability sampling	Probabilistic	Probabilistic

#### Populations and regions

Table 3 presents all studies by study populations and category of method (see also Additional file 1: Table S1). About 77 % (*n* = 205) of the retrieved studies referred to MSM population, FSWs were subject in about 18 % (*n* = 48), MSWs were included in 1.5 % (*n* = 4) and TSWs were identified in 0.4 % (*n* = 1) publications. The remaining 3.7 % of the studies related to more than one (sub) population: four studies were related to MSM/TG, three studies presented information about MSM/MSW/TG and FSW/MSM, MSM/MSW and MSM/MSW/TSW were referred in one study each. About 83 % (*n* = 49) of sex work publications were female sex work related.

**Table 3:** Study populations by categories

Populations	Non-probabilistic *N* (%)	Semi-probabilistic *N* (%)	Probabilistic *N* (%)	Mixed^a^ *N* (%)	Total *N* (%)	References^b^
MSM	64 (23.9)	128 (47.8)	6 (2.2)	7 (2.6)	205 (76.5)	[1–5,7,78–80,82–84,120–126,153,155,156,158–160,189–194,222,223,237,255] [11–13,15–18,20,85,87–89,127,128,131–135,162–164,167,168,196–200,224–226,228,243,253,254,256,260,264,267] [22–25,29–31,35–40,90–104,137,138,169–171,175,176,178,201–204,206–209,229,230,244,250,265,266] [42–50,52,53,56–58,60–62,105–107,109,111–113,139,143–147,149,150,179–181,183,184,210–218,245,246,259,261] [64,67–76,115,116,118,119,152,185–188,219–221,236,242,258,263,268]
FSW	24 (9.0)	21 (7.8)	3 (1.1)	0	48 (17.9)	[8–10,14,19,21,26–28,33,34,41,51,54,55,59,63,66,77,117,129,136,140,142,151,157,161,165–167,172–174,177,182,227,231–235,239,241,247–249,251,262]
MSW	2 (0.7)	2 (0.7)	0	0	4 (1.5)	[108,154,205,252]
TSW	1 (0.4)	0	0	0	1 (0.4)	[148]
MSM/TG	3 (1.1)	1 (0.4)	0	0	3 (1.5)	[6,141,238,240]
FSW/MSM	0	1 (0.4)	0	0	1 (0.4)	[32]
MSM/MSW	0	1 (0.4)	0	0	1 (0.4)	[114]
MSM/MSW/TG	0	3 (1.1)	0	0	3 (1.1)	[81,86,110]
MSM/MSW/TSW	0	0	0	1 (0.4)	1 (0.4)	[257]
Total	94 (35.1)	157 (58.6)	9 (3.4)	8 (3.0)	268 (100.0)	

^a^Publications that mentioned at least two categories of recruitment methods

^b^References can be found in Additional file 4: Text S3

Our search found that FSWs are more frequently recruited by non-probabilistic methods than MSM (odds = 2.2; *p* < 0.05; CI [1.1–4.2]). Also, we found that the *semi*-*probabilistic* methods were mainly used in studies of men who have sex with men.

We identified 53 countries and regions (Additional file 2: Table S2) as being the origin of the identified studies. Countries were classified into eight regions (Table 4). More than 70 % of the studies were published in the regions of North America (40.7 %, *n* = 107) and Asia and Pacific (30 %, *n* = 81). North Africa and Middle East region was the region that published fewer studies (0.8 %, *n* = 2).

**Table 4:** Publications by region

Regions	Countries (*N*)	Publications (%)
Asia and Pacific	12	81 (30.8)
East and Southern Africa	7	20 (7.6)
Eastern Europe and Central Asia	7	11 (4.2)
Latin America	7	21 (8.0)
North Africa and Middle East	2	2 (0.8)
North America	2	107 (40.7)
West and Central Africa	4	8 (3.0)
West and Central Europe	7	13 (4.9)
Total	48	263

Five publications were excluded from belonging to more than one region

Although most of the retrieved publications belong to the VHHHD regions (58 %), our review found that, in the last 6 years, the number of studies published based in MLHD countries increased more than those based in VHHHD countries (odds = 2.541; *p* < 0.05; CI [1.327–4.866]) (Table 5).

**Table 5:** Populations and years of publication by region’s development level

Years	MLHD countries (%)	VHHHD countries (%)	Total
2003–2007	15 (29.4)	36 (70.6)	51
2008–2013	95 (44.8)	117 (55.2)	212

Five publications were excluded from belonging to more than one region

## Discussion

This is to our knowledge the first systematic literature review of methods used to sample most-at-risk populations of FSW and MSM. We identified 268 published articles from 53 countries or regions and 11 recruitment methods. Over 427,000 participants were surveyed in these 268 studies. Sampling methods we classified as semi-probabilistic (internet, TLS and RDS) were used in 59 % of the retrieved studies. These results are consistent with prior studies finding that web-based surveys, TLS and RDS methods have been used more extensively in health research in the past years [28–30]. The increase in the use of semi-probabilistic methods might be associated with the 2005 proposal of United Nations General Assembly Special Session^2^ (UNGASS) that proposed a new set of indicators according to the level of epidemics in countries: generalized or concentrated [31]. Probabilistic methods were applied in less than 5 % of the studies, which may be because implementing a probabilistic approach in most-at-risk populations is expensive, inconvenient or impossible [2].

Our systematic literature review found that FSWs were more frequently recruited by non-probabilistic methods than were MSM, who were more often recruited with semi-probabilistic methods. The WHO suggests that the sampling strategies used to collect data on MSM should be RDS, TLS or cluster sampling while to collect data on FSW, TLS or convenience sampling should be used [2]. Our results are thus consistent with WHO suggestions and also in accordance with other studies that found FSW being mainly recruited by non-probabilistic methods [32] and MSM being primarily recruited by RDS, Internet and TLS [33, 34] methods. *Semi*-*probabilistic* methods, namely RDS and Internet, require that target populations form some kind of social network [35]. Some studies reported that FSW usually have smaller peer network groups than other high-risk groups [36, 37], and they also have few friends/close friends among recruiters [36], which might explain why this population is mainly recruited by non-probabilistic methods.

The aid to MLHD countries on STD control has increased about 75 % between 2008 and 2013 when compared to the period of 2003–2007 [11]. This additional aid might be an explanation for the increasing number of publications once the number of published articles can be seen as an indicator of productivity [38].

Retrieved publications that employed *semi*-*probabilistic* methods gathered the greatest number of respondents. These methods have the advantage of reaching the most “hidden” individuals among “hidden” populations [32], which is a gain not only when compared to non-probabilistic methods but also when compared with probabilistic ones that always demand for a sampling frame and can miss many hidden individuals [1].

Providing unbiased estimates of prevalence for HIV surveillance is crucial for effective public health interventions [2]. However, reliable estimates cannot be produced without an appropriate sampling approach, which depends on several factors including the local context, availability of resources and the target population. Thus, the same method is not necessarily the best for all situations, populations and countries [34].

In practice, researchers studying HRP behaviours have to choose between several sampling methods, and there are no precise guidelines in the literature to choose one method over another. Moreover, in this field, systematic reviews are particularly challenging because public health problems require us to draw on complex sets of quantitative evidence [39]. This study provides researchers working in this area with a systematic evaluation of the sampling methods used by other researchers and may be especially useful for readers and other investigators who consider new research projects that address hard to reach populations.

### Limitations

This study has several limitations. One limitation is the definition of inclusion and exclusion criteria; searching for published studies only might have left behind many studies. Additionally, searching just for English written studies also leaves out studies published in other languages, which might be relevant for our purpose. Having the HRP’s name in the publication title may have excluded some potentially eligible studies from this review. Another limitation concerns the identification of populations and sub populations: we searched for MSM term in the title only which might have left out of this review several (sub) populations such as transgender or bisexual persons. Lastly, several publications were identified using the same database, hence the same recruitment strategy, which might have led to biased results.

## Conclusions

This systematic literature review found that 11 methods had been used to sample MSM and FSW. These 11 methods were categorized in three categories. The semi-probabilistic category was the most commonly used method to survey MSM, and the Internet was the method that gathered more respondents. Female sex workers were mainly recruited by non-probabilistic methods. Most published studies originated in the regions of North America and Asia Pacific. While most of the retrieved publications belong to the VHHHD regions, in the last 6 years, the number of studies published based in MLHD countries has been increasing.

### Endnotes

^1^http://www.b-on.pt/en/.

^2^The UNGASS is a meeting of the United Nations member states to assess and discuss global topics such as health, gender or drugs priorities. In 2005, a declaration of commitment of member states was produced as a result from the UNGASS meeting in 2001. This declaration of commitment is the result of the global consensus that member states reached in order to achieve the Millennium Development Goals of halting and reversing the HIV epidemics. Several key indicators were then proposed to measure the effectiveness of the response of each country in fighting HIV (http://data.unaids.org/publications/irc-pub06/jc1126-constrcoreindic-ungass_en.pdf)

## Additional files

Additional file 1: Table S1. Recruitment methods by population type. Additional file 2: Table S2. Country/region of origin by population type. Additional file 3: Text S2. PRISMA c121hecklist. Additional file 4: Text S3. List of retrieved publications analysed in the systematic literature review. Additional file 5: Text S4. Sampling methods identified in the systematic literature review.
