# Supplementary material for: Hard-to-reach populations of men who have sex with men and sex workers: a systematic review on sampling methods
Source: Syst Rev. 2015 Oct 30;4:141. doi: 10.1186/s13643-015-0129-9 (PMC4627393; doi:10.1186/s13643-015-0129-9)
Supplement: Additional file 1: Table S1. — Recruitment methods by population type. [file 13643_2015_129_MOESM1_ESM.pdf]

Table S1: Recruitment Methods by Population Type

Method	MSM (%)	FSW (%)	MSW (%)	TSW (%)	MSM/TG (%)	FSW/MSM (%)	MSM/MSW (%)	MSM/MSW/TG (%)	MSM/MSW/TSW (%)	Total (%)
RDS	56 (20.9)	19 (7.1)	0	0	1 (0.4)	1 (0.4)	0	0	0	77 (28.7)
TLS	36 (13.4)	1 (0.4)	1 (0.4)	0	0	0	1 (0.4)	3 (1.1)	0	42 (15.7)
Convenience	26 (9.7)	5 (1.9)	0	1 (0.4)	1 (0.4)	0	0	0	0	33 (12.3)
Snowball	25 (9.3)	10 (3.7)	1 (0.4)	0	0	0	0	0	0	36 (13.4)
Internet	32 (11.9)	0	1 (0.4)	0	0	0	0	0	0	33 (12.3)
Targeted	9 (3.4)	6 (2.2)	0	0	0	0	0	0	0	15 (5.6)
Purposive	2 (0.7)	2 (0.7)	0	0	2 (0.7)	0	0	0	0	6 (2.2)
Multi-stage probability sample	4 (1.5)	0	0	0	0	0	0	0	0	4 (1.5)
Cluster	0	3 (1.1)	0	0	0	0	0	0	0	3 (1.1)
Convenience, Snowball	1 (0.4)	1 (0.4)	1 (0.4)	0	0	0	0	0	0	3 (1.1)
TLS, Internet	2 (0.7)	0	0	0	0	0	0	0	0	2 (0.7)
RDD	2 (0.7)	0	0	0	0	0	0	0	0	2 (0.7)
Convenience, Internet	1 (0.4)	0	0	0	0	0	0	0	1 (0.4)	2 (0.7)

Convenience, RDD	1 (0.4)	0	0	0	0	0	0	0	0	1 (0.4)
Convenience, RDS	1 (0.4)	0	0	0	0	0	0	0	0	1 (0.4)
Internet, Snowball	1 (0.4)	0	0	0	0	0	0	0	0	1 (0.4)
RDS, TLS	1 (0.4)	1 (0.4)	0	0	0	0	0	0	0	2 (0.7)
Stratified probability sampling, Internet	1 (0.4)	0	0	0	0	0	0	0	0	1 (0.4)
Targeted, Snowball	1 (0.4)	0	0	0	0	0	0	0	0	1 (0.4)
Convenience, Snowball, Internet	1 (0.4)	0	0	0	0	0	0	0	0	1 (0.4)
Snowball, TLS, RDS	1 (0.4)	0	0	0	0	0	0	0	0	1 (0.4)
RDS, Internet	1 (0.4)	0	0	0	0	0	0	0	0	1 (0.4)
**Total**	**205 (76.5)**	**48 (17.9)**	**4 (1.5)**	**1 (0.4)**	**4 (1.5)**	**1 (0.4)**	**1 (0.4)**	**3 (1.1)**	**1 (0.4)**	**268**
