# Supplementary material for: Hard-to-reach populations of men who have sex with men and sex workers: a systematic review on sampling methods
Source: Syst Rev. 2015 Oct 30;4:141. doi: 10.1186/s13643-015-0129-9 (PMC4627393; doi:10.1186/s13643-015-0129-9)
Supplement: Additional file 2: Table S2. — Country/region of origin by population type. [file 13643_2015_129_MOESM2_ESM.pdf]

Table S2: Country/Region of the studies by population type

Country	MSM	FSW	MSW	TSW	MSM/TG	FSW/MSM	MSM/MSW	MSM/MSW/TG	MSM/MSW/TSW	Total (%)	References
Afghanistan	0	1	0	0	0	0	0	0	0	1 (0.4)	[151]
Argentina	4	0	0	0	0	0	0	0	0	4 (1.5)	[7,40,69,183]
Asia	1	0	0	0	0	0	0	0	0	1 (0.4)	[203]
Australia	1	1	0	0	0	0	0	0	0	2 (0.8)	[140,207]
Benin, Guinea, Senegal	0	1	0	0	0	0	0	0	0	1 (0.4)	[129]
Brasil	5	3	0	0	1	0	0	0	0	9 (3.4)	[2,6,8,9,39,54,72,160,267]
Cameroon	2	0	0	0	0	0	0	0	0	2 (0.8)	[70,171]
Canada	2	0	0	0	0	0	0	0	0	2 (0.8)	[100,145]
China	31	14	1	0	0	0	1	0	0	47 (17.5)	[12,15,26–31,58–62,75,76,103,114,130,132,147,149,150,158,159,161,162,165–170,172,177,179,184,186,188,212,218,241,247,249,252,260–262]
Croatia	4	0	0	0	0	0	0	0	0	4 (1.5)	[4,5,25,180]
Croatia, Montenegro	0	1	0	0	0	0	0	0	0	1 (0.4)	[251]
El Salvador	1	0	0	0	0	0	0	0	0	1 (0.4)	[64]
Estonia	1	1	0	0	0	0	0	0	0	2 (0.8)	[55,146]
Europe	2	0	0	0	0	0	0	0	0	2 (0.8)	[126,219]
Gambia	1	0	0	0	0	0	0	0	0	1 (0.4)	[187]
Germany	1	0	0	0	0	0	0	0	0	1 (0.4)	[204]
Guatemala	1	0	0	0	1	0	0	0	0	2 (0.8)	[238,263]
Honduras	0	1	0	0	0	0	0	0	0	1 (0.4)	[21]

India	5	8	0	0	1	0	0	0	0	14 (5.2)	[10,14,33,34,41,51,53,63,102, 133,139,141,181,239]
Indonesia	0	0	0	0	0	0	0	0	1	1 (0.4)	[257]
Israel	1	1	0	0	0	0	0	0	0	2 (0.8)	[136,138]
Japan	1	0	0	0	0	0	0	0	0	1 (0.4)	[197]
Kazakhstan	1	0	0	0	0	0	0	0	0	1 (0.4)	[3]
Kenya	1	3	0	0	0	0	0	0	0	4 (1.5)	[157,173–175]
Lao	1	0	0	0	0	0	0	0	0	1 (0.4)	[105]
Lebanon	0	0	0	0	0	1	0	0	0	1 (0.4)	[32]
Malawi	1	0	0	0	0	0	0	0	0	1 (0.4)	[73]
Malawi, Namibia, Botswana	1	0	0	0	0	0	0	0	0	1 (0.4)	[155]
Malaysia	1	0	0	0	0	0	0	0	0	1 (0.4)	[90]
Mexico	1	1	0	0	0	0	0	0	0	2 (0.8)	[121,142]
Moldova	0	1	0	0	0	0	0	0	0	1 (0.4)	[77]
Mongolia	1	0	0	0	0	0	0	0	0	1 (0.4)	[74]
Netherlands	3	0	0	0	0	0	0	0	0	3 (1.1)	[189,217,222]
Nigeria	4	0	0	0	0	0	0	0	0	4 (1.5)	[1,143,185,237]
Peru	2	0	0	0	0	0	0	0	0	2 (0.8)	[115,123]
Russia	0	0	1	0	0	0	0	0	0	1 (0.4)	[154]
Russia, USA	1	0	0	0	0	0	0	0	0	1 (0.4)	[17]
Singapore	1	0	0	0	0	0	0	0	0	1 (0.4)	[268]
South Africa	9	1	0	0	0	0	0	0	0	10 (3.7)	[22,48,49,56,78,152,153,176,213,235]
South Korea	0	2	0	0	0	0	0	0	0	2 (0.8)	[117,248]
Spain	1	0	0	0	0	0	0	0	0	1 (0.4)	[128]

Swaziland	1	0	0	0	0	0	0	0	0	1 (0.4)	[71]
Sweden	1	0	0	0	0	0	0	0	0	1 (0.4)	[210]
Switzerland	1	0	0	0	0	0	0	0	0	1 (0.4)	[120]
Tanzania (Zanzibar)	1	0	0	0	0	0	0	0	0	1 (0.4)	[20]
Thailand	3	1	1	0	0	0	0	3	0	8 (3.0)	[66,81,86,97,98,108–110]
Uganda	2	0	0	0	0	0	0	0	0	2 (0.8)	[16,65]
UK	5	0	0	0	0	0	0	0	0	5 (1.9)	[11,144,195,258,264]
USA	95	5	1	1	1	0	0	0	0	103 (38.4)	[13,79,80,82–85,87,122,124,125,127,131,156,163,164,190–194,196,198,199,223–228,243,253–256][18,23,24,35–38,88,89,91–95,99,134,135,137,200–202,205,206,208,229,244,266] [42-47,50,52,57,67,68,101,104,106, 107,111,113,116,118,119,148,178,209, 211,214,216,220,221,230–234,236,240,245,246,250,259,265]
USA, Puerto Rico	1	0	0	0	0	0	0	0	0	1 (0.4)	[96]
USA, Canada	1	0	0	0	0	0	0	0	0	1 (0.4)	[215]
Uzbekistan	0	1	0	0	0	0	0	0	0	1 (0.4)	[182]
Vietnam	1	1	0	0	0	0	0	0	0	2 (0.8)	[19,242]
**Total**	**205**	**48**	**4**	**1**	**4**	**1**	**1**	**3**	**1**	**268 (100)**	
