# Supplementary material for: Hard-to-reach populations of men who have sex with men and sex workers: a systematic review on sampling methods
Source: Syst Rev. 2015 Oct 30;4:141. doi: 10.1186/s13643-015-0129-9 (PMC4627393; doi:10.1186/s13643-015-0129-9)
Supplement: Additional file 4: Text S3. — List of retrieved publications analysed in the systematic literature review. [file 13643_2015_129_MOESM4_ESM.pdf]

Text S3: List of retrieved publications analysed in the systematic review

1. Adebajo SB, Eluwa GI, Allman D, Myers T, Ahonsi BA: **Prevalence of internalized homophobia and HIV associated risks among men who have sex with men in Nigeria**. Afr J Reprod Health 2012, 16: 21-28.

2. Bermudez-Aza EH, Kerr LRFS, Kendall C, Pinho AA, de Mello MB, Mota RS, Guimaraes MDC, Alencar CS, de Brito AM, Dourado IC, da Batista SMB, Abreu F, de Oliveira LC, Moraes AD, Benzaken AS, Merchan-Hamann E, de Freitas GMB, McFarland W, Albuquerque E, Rutherford GW, Sabino E: **Antiretroviral Drug Resistance in a Respondent-Driven Sample of HIV-Infected Men Who Have Sex With Men in Brazil**. Jaids-Journal of Acquired Immune Deficiency Syndromes 2011, 57: S186-S192.

3. Berry M, Wirtz AL, Janayeva A, Ragoza V, Terlikbayeva A, Amirov B, Baral S, Beyrer C: **Risk Factors for HIV and Unprotected Anal Intercourse among Men Who Have Sex with Men (MSM) in Almaty, Kazakhstan**. Plos One 2012, 7:

4. Bozicevic I, Lepej SZ, Rode OD, Grgic I, Jankovic P, Dominkovic Z, Lukas D, Johnston LG, Begovac J: **Prevalence of HIV and sexually transmitted infections and patterns of recent HIV testing among men who have sex with men in Zagreb, Croatia**. Sexually Transmitted Infections 2012, 88: 539-544.

5. Bozicevic I, Rode OD, Lepej SZ, Johnston LG, Stulhofer A, Dominkovic Z, Bacak V, Lukas D, Begovac J: **Prevalence of Sexually Transmitted Infections Among Men Who Have Sex with Men in Zagreb, Croatia**. Aids and Behavior 2009, 13: 303-309.

6. Carballo-Dieguez A, Balan I, Dolezal C, Mello MB: **Recalled Sexual Experiences in Childhood with Older Partners: A Study of Brazilian Men Who Have Sex with Men and Male-to-Female Transgender Persons**. Archives of Sexual Behavior 2012, 41: 363-376.

7. Carballo-Dieguez A, Balan I, Marone R, Pando MA, Dolezal C, Barreda V, Leu CS, Avila MM: **Use of Respondent Driven Sampling (RDS) Generates a Very Diverse Sample of Men Who Have Sex with Men (MSM) in Buenos Aires, Argentina**. Plos One 2011, 6:

8. Damacena GN, Szwarcwald CL, Barbosa A: **Implementation of respondent-driven sampling among female sex workers in Brazil, 2009**. Cadernos de Saude Publica 2011, 27: S45-S55.

9. Damacena GN, Szwarcwald CL, de Souza PRB, Dourado I: **Risk Factors Associated With HIV Prevalence Among Female Sex Workers in 10 Brazilian Cities**. Jaids-Journal of Acquired Immune Deficiency Syndromes 2011, 57: S144-S152.

10. Erausquin JT, Reed E, Blankenship KM: **Police-Related Experiences and HIV Risk Among Female Sex Workers in Andhra Pradesh, India**. Journal of Infectious Diseases 2011, 204: S1223-S1228.

11. Evans AR, Hart GJ, Mole R, Mercer CH, Parutis V, Gerry CJ, Imrie J, Burns FM: **Central and East European migrant men who have sex with men in London: a comparison of recruitment methods**. Bmc Medical Research Methodology 2011, 11:

12. Fan S, Lu HY, Ma XY, Sun YM, He X, Li CM, Raymond HF, McFarland W, Sun JP, Ma W, Jia YJ, Xiao Y, Shao YM, Ruan YH: **Behavioral and Serologic Survey of Men Who Have Sex with Men in Beijing, China: Implication for HIV Intervention**. Aids Patient Care and Stds 2012, 26: 148-155.

13. Gorbach PM, Murphy R, Weiss RE, Hucks-Ortiz C, Shoptaw S: **Bridging Sexual Boundaries: Men Who Have Sex with Men and Women in a Street-Based Sample in Los Angeles**. Journal of Urban Health-Bulletin of the New York Academy of Medicine 2009, 86: S63-S76.

14. Gupta J, Reed E, Kershaw T, Blankenship KM: **History of sex trafficking, recent experiences of violence, and HIV vulnerability among female sex workers in coastal Andhra Pradesh, India**. International Journal of Gynecology & Obstetrics 2011, 114: 101-105.

15. Hao C, Huan XP, Yan HJ, Yang HT, Guan WH, Xu XQ, Zhang M, Wang N, Tang WM, Gu J, Lau JTF: **A Randomized Controlled Trial to Evaluate the Relative Efficacy of Enhanced Versus Standard Voluntary Counseling and Testing on Promoting Condom Use among Men Who Have Sex with Men in China**. Aids and Behavior 2012, 16: 1138-1147.

16. Hladik W, Barker J, Ssenkusu JM, Opio A, Tappero JW, Hakim A, Serwadda D, Grp CS: **HIV Infection among Men Who Have Sex with Men in Kampala, Uganda-A Respondent Driven Sampling Survey**. Plos One 2012, 7:

17. Iguchi MY, Ober AJ, Berry SH, Fain T, Heckathorn DD, Gorbach PM, Heimer R, Kozlov A, Ouellet LJ, Shoptaw S, Zule WA: **Simultaneous Recruitment of Drug Users and Men Who Have Sex with Men in the United States and Russia Using Respondent-Driven Sampling: Sampling Methods and Implications**. Journal of Urban Health-Bulletin of the New York Academy of Medicine 2009, 86: S5-S31.

18. Johnson CV, Mimiaga MJ, Reisner SL, Tetu AM, Cranston K, Bertrand T, Novak DS, Mayer KH: **Health Care Access and Sexually Transmitted Infection Screening Frequency Among At-Risk Massachusetts Men Who Have Sex With Men**. American Journal of Public Health 2009, 99: S187-S192.

19. Johnston LG, Sabin K, Hien MT, Huong PT: **Assessment of respondent driven sampling for recruiting female sex workers in two Vietnamese cities: Reaching the unseen sex worker**. Journal of Urban Health-Bulletin of the New York Academy of Medicine 2006, 83: I16-I28.

20. Johnston LG, Holman A, Dahoma M, Miller LA, Kim E, Mussa M, Othman AA, Kim A, Kendall C, Sabin K: **HIV risk and the overlap of injecting drug use and high-risk sexual behaviours among men who have sex with men in Zanzibar (Unguja), Tanzania**. International Journal of Drug Policy 2010, 21: 485-492.

21. Johnston LG, Paz-Bailey G, Morales-Miranda S, Morgan M, Alvarez B, Hickman L, Monterroso E: **High prevalence of Mycoplasma genitalium among female sex workers in Honduras: implications for the spread of HIV and other sexually transmitted infections**. International Journal of Std & Aids 2012, 23: 5-11.

22. Lane T, Raymond HF, Dladla S, Rasethe J, Struthers H, McFarland W, McIntyre J: **High HIV Prevalence Among Men Who have Sex with Men in Soweto, South Africa: Results from the Soweto Men's Study**. Aids and Behavior 2011, 15: 626-634.

23. Lauby JL, Marks G, Bingham T, Liu KL, Liau A, Stueve A, Millett GA: **Having Supportive Social Relationships is Associated with Reduced Risk of Unrecognized HIV Infection Among Black and Latino Men who Have Sex with Men**. Aids and Behavior 2012, 16: 508-515.

24. Lauby JL, Millett GA, LaPollo AB, Bond L, Murrill CS, Marks G: **Sexual risk behaviors of HIV-positive, HIV-negative, and serostatus-unknown Black men who have sex with men and women**. Archives of Sexual Behavior 2008, 37: 708-719.

25. Lepej SZ, Vrakela IB, Poljak M, Bozicevic I, Begovac J: **Phylogenetic Analysis of HIV Sequences Obtained in a Respondent-Driven Sampling Study of Men Who Have Sex with Men**. Aids Research and Human Retroviruses 2009, 25: 1335-1338.

26. Li J, Chen XS, Merli MG, Weir SS, Henderson GE: **Systematic Differences in Risk Behaviors and Syphilis Prevalence Across Types of Female Sex Workers: A Preliminary Study in Liuzhou, China**. Sexually Transmitted Diseases 2012, 39: 195-200.

27. Li Y, Detels R, Lin P, Fu XB, Deng ZM, Liu YY, Huang GH, Li J, Tan YH: **Difference in Risk Behaviors and STD Prevalence Between Street-Based and Establishment-Based FSWs in Guangdong Province, China**. Aids and Behavior 2012, 16: 943-951.

28. Liao MZ, Nie XJ, Pan RJ, Wang CX, Ruan SM, Zhang CQ, Kang DM, Fu JH, Qian YS, Tao XR, Zhao JK: **Consistently Low Prevalence of Syphilis among Female Sex Workers in Jinan, China: Findings from Two Consecutive Respondent Driven Sampling Surveys**. Plos One 2012, 7:

29. Liu HJ, Feng TJ, Liu H, Feng HC, Cai YM, Rhodes AG, Grusky O: **Egocentric Networks of Chinese Men Who Have Sex with Men: Network Components, Condom Use Norms, and Safer Sex**. Aids Patient Care and Stds 2009, 23: 885-893.

30. Liu J, Qu B, Guo HQ, Sun G: **Factors That Influence Risky Sexual Behaviors Among Men Who Have Sex with Men in Liaoning Province, China: A Structural Equation Model**. Aids Patient Care and Stds 2011, 25: 423-429.

31. Ma XY, Zhang QY, He X, Sun WD, Yue H, Chen S, Raymond HF, Li Y, Xu M, Du H, McFarland W: **Trends in prevalence of HIV, syphilis, hepatitis C, hepatitis B, and sexual risk behavior among men who have sex with men - Results of 3 consecutive respondent-driven sampling surveys in Beijing, 2004 through 2006**. Jaids-Journal of Acquired Immune Deficiency Syndromes 2007, 45: 581-587.

32. Mahfoud Z, Afifi R, Ramia S, El Khoury D, Kassak K, El Barbir F, Ghanem M, El-Nakib M, DeJong J: **HIV/AIDS among female sex workers, injecting drug users and men who have sex with men in Lebanon: results of the first biobehavioral surveys**. Aids 2010, 24: S45-S54.

33. Medhi GK, Mahanta J, Kermode M, Paranjape RS, Adhikary R, Phukan SK, Ngully P: **Factors associated with history of drug use among female sex workers (FSW) in a high HIV prevalence state of India**. Bmc Public Health 2012, 12:

34. Medhi GK, Mahanta J, Paranjape RS, Adhikary R, Laskar N, Ngully P: **Factors associated with HIV among female sex workers in a high HIV prevalent state of India**. Aids Care-Psychological and Socio-Medical Aspects of Aids/Hiv 2012, 24: 369-376.

35. Millett GA, Ding H, Lauby J, Flores S, Stueve A, Bingham T, Carballo-Dieguez A, Murrill C, Liu KL, Wheeler D, Liau A, Marks G: **Circumcision status and HIV infection among black and Latino men who have sex with men in 3 US cities**. Jaids-Journal of Acquired Immune Deficiency Syndromes 2007, 46: 643-650.

36. Mimiaga MJ, Goldhammer H, Belanoff C, Tetu AM, Mayer KH: **Men who have sex with men: Perceptions about sexual risk, HIV and sexually transmitted disease testing, and provider communication**. Sexually Transmitted Diseases 2007, 34: 113-119.

37. Mimiaga MJ, Reisner SL, Bland S, Skeer M, Cranston K, Isenberg D, Vega BA, Mayer KH: **Health System and Personal Barriers Resulting in Decreased Utilization of HIV and STD Testing Services among At-Risk Black Men Who Have Sex with Men in Massachusetts**. Aids Patient Care and Stds 2009, 23: 825-835.

38. Mizuno Y, Borkowf C, Millett GA, Bingham T, Ayala G, Stueve A: **Homophobia and Racism Experienced by Latino Men Who Have Sex with Men in the United States: Correlates of Exposure and Associations with HIV Risk Behaviors**. Aids and Behavior 2012, 16: 724-735.

39. Mota RMS, Kerr LRFS, Kendall C, Pinho A, de Mello MB, Dourado I, Guimaraes MDC, Brito A, Batista S, Abreu F, Benzaken A, Oliveira L, Moraes A, Merchan-Hamann E, Freitas G, Albuquerque EM, McFarland W, Rutherford G: **Reliability of Self-Report of HIV Status Among Men Who Have Sex With Men in Brazil**. Jaids-Journal of Acquired Immune Deficiency Syndromes 2011, 57: S153-S156.

40. Pando MA, Balan IC, Marone R, Dolezal C, Leu CS, Squiquera L, Barreda V, Fermepin MR, Vaulet LG, Rey J, Picconi M, Carballo-Dieguez A, Avila MM: **HIV and Other Sexually Transmitted Infections among Men Who Have Sex with Men Recruited by RDS in Buenos Aires, Argentina: High HIV and HPV Infection**. Plos One 2012, 7:

41. Reed E, Gupta J, Biradavolu M, Blankenship KM: **Migration/mobility and risk factors for HIV among female sex workers in Andhra Pradesh, India: implications for HIV prevention**. Int J STD AIDS 2012, 23: e7-e13.

42. Reisner SL, Mimiaga MJ, Case P, Johnson CV, Safren SA, Mayer KH: **Predictors of Identifying as a Barebacker among High-Risk New England HIV Seronegative Men Who Have Sex with Men**. Journal of Urban Health-Bulletin of the New York Academy of Medicine 2009, 86: 250-262.

43. Reisner SL, Mimiaga MJ, Johnson CV, Bland S, Case P, Safren SA, Mayer KH: **What Makes a Respondent-Driven Sampling "Seed" Productive? Example of Finding At-Risk Massachusetts Men Who Have Sex with Men**. Journal of Urban Health-Bulletin of the New York Academy of Medicine 2010, 87: 467-479.

44. Reisner SL, Mimiaga MJ, Bland S, Skeer M, Cranston K, Isenberg D, Driscoll M, Mayer KH: **Problematic alcohol use and HIV risk among Black men who have sex with men in Massachusetts**. Aids Care-Psychological and Socio-Medical Aspects of Aids/Hiv 2010, 22: 577-587.

45. Reisner SL, Mimiaga MJ, Safren SA, Mayer KH: **Stressful or traumatic life events, post-traumatic stress disorder (PTSD) symptoms, and HIV sexual risk taking among men who have sex with men**. Aids Care-Psychological and Socio-Medical Aspects of Aids/Hiv 2009, 21: 1481-1489.

46. Reisner SL, Mimiaga MJ, Skeer M, Bright D, Cranston K, Isenberg D, Bland S, Barker TA, Mayer KH: **Clinically Significant Depressive Symptoms as a Risk Factor for HIV Infection Among Black MSM in Massachusetts**. Aids and Behavior 2009, 13: 798-810.

47. Rhodes SD, Mccoy TP, Hergenrather KC, Vissman AT, Wolfson M, Alonzo J, Bloom FR, Alegria-Ortega J, Eng E: **Prevalence Estimates of Health Risk Behaviors of Immigrant Latino Men Who Have Sex With Men**. Journal of Rural Health 2012, 28: 73-83.

48. Rispel LC, Metcalf CA, Cloete A, Moorman J, Reddy V: **You become afraid to tell them that you are gay: Health service utilization by men who have sex with men in South African cities**. Journal of Public Health Policy 2011, 32: S137-S151.

49. Rispel LC, Metcalf CA, Cloete A, Reddy V, Lombard C: **HIV Prevalence and Risk Practices Among Men Who Have Sex With Men in Two South African Cities**. Jaids-Journal of Acquired Immune Deficiency Syndromes 2011, 57: 69-76.

50. Schneider JA, Walsh T, Cornwell B, Ostrow D, Michaels S, Laumann EO: **HIV Health Center Affiliation Networks of Black Men Who Have Sex With Men: Disentangling Fragmented Patterns of HIV Prevention Service Utilization**. Sexually Transmitted Diseases 2012, 39: 598-604.

51. Shahmanesh M, Wayal S, Cowan F, Mabey D, Copas A, Patel V: **Suicidal Behavior Among Female Sex Workers in Goa, India: The Silent Epidemic**. American Journal of Public Health 2009, 99: 1239-1246.

52. Shoptaw S, Weiss RE, Munjas B, Hucks-Ortiz C, Young SD, Larkins S, Victorianne GD, Gorbach PM: **Homonegativity, Substance Use, Sexual Risk Behaviors, and HIV Status in Poor and Ethnic Men Who Have Sex with Men in Los Angeles**. Journal of Urban Health-Bulletin of the New York Academy of Medicine 2009, 86: S77-S92.

53. Solomon SS, Srikrishnan AK, Sifakis F, Mehta SH, Vasudevan CK, Balakrishnan P, Mayer KH, Solomon S, Celentano DD: **The Emerging HIV Epidemic among Men Who have Sex with Men in Tamil Nadu, India: Geographic Diffusion and Bisexual Concurrency**. Aids and Behavior 2010, 14: 1001-1010.

54. Szwarcwald CL, de Souza PRB, Damacena GN, Barbosa AB, Kendall C: **Analysis of Data Collected by RDS Among Sex Workers in 10 Brazilian Cities, 2009: Estimation of the Prevalence of HIV, Variance, and Design Effect**. Jaids-Journal of Acquired Immune Deficiency Syndromes 2011, 57: S129-S135.

55. Uuskula A, Johnston LG, Raag M, Trummal A, Talu A, Des Jarlais DC: **Evaluating Recruitment among Female Sex Workers and Injecting Drug Users at Risk for HIV Using Respondent-driven Sampling in Estonia**. Journal of Urban Health-Bulletin of the New York Academy of Medicine 2010, 87: 304-317.

56. Vu L, Tun W, Sheehy M, Nel D: **Levels and Correlates of Internalized Homophobia Among Men Who Have Sex with Men in Pretoria, South Africa**. Aids and Behavior 2012, 16: 717-723.

57. Wheeler DP, Lauby JL, Liu KL, Van Sluytman LG, Murrill C: **A comparative analysis of sexual risk characteristics of Black men who have sex with men or with men and women**. Archives of Sexual Behavior 2008, 37: 697-707.

58. Yan HJ, Yang HT, Zhao JK, Wei CY, Li JJ, Huan XP, Zhang M, Raymond HF, McFarland W: **Long-Chain Peer Referral of Men Who Have Sex With Men: A Novel Approach to Establish and Maintain a Cohort to Measure HIV Incidence, Nanjing, China**. Jaids-Journal of Acquired Immune Deficiency Syndromes 2012, 59: 177-184.

59. Zhang H, Liao MZ, Nie XJ, Pan RJ, Wang CX, Ruan SM, Zhang CQ, Tao XR, Kang DM, Jiang BF: **Predictors of consistent condom use based on the Information-Motivation-Behavioral Skills (IMB) model among female sex workers in Jinan, China**. Bmc Public Health 2011, 11:

60. Zhang HB, Wu ZY, Zheng YJ, Wang J, Zhu JL, Xu J: **A Pilot Intervention to Increase Condom Use and HIV Testing and Counseling Among Men Who Have Sex With Men in Anhui, China**. Jaids-Journal of Acquired Immune Deficiency Syndromes 2010, 53: S88-S92.

61. Zhang L, Ding XB, Lu RR, Feng LG, Li XF, Xiao Y, Ruan YH, Vermund SH, Shao YM, Qian HZ: **Predictors of HIV and Syphilis among Men Who Have Sex with Men in a Chinese Metropolitan City: Comparison of Risks among Students and Non-Students**. Plos One 2012, 7:

62. Zhong F, Lin P, Xu HF, Wang Y, Wang M, He Q, Fan LR, Li Y, Wen F, Liang YR, Raymond HF, Zhao JK: **Possible Increase in HIV and Syphilis Prevalence Among Men Who Have Sex with Men in Guangzhou, China: Results from a Respondent-Driven Sampling Survey**. Aids and Behavior 2011, 15: 1058-1066.

63. Armstrong G, Medhi GK, Kermode M, Mahanta J, Goswami P, Paranjape R: **Exposure to HIV prevention programmes associated with improved condom use and uptake of HIV testing by female sex workers in Nagaland, Northeast India**. Bmc Public Health 2013, 13: 476

64. Kim EJ, Creswell J, Guardado ME, Shah N, Kim AA, Nieto AI, Hernandez-Ayala FD, Monterroso E, Paz-Bailey G: **Correlates of Bisexual Behaviors Among Men who have Sex with Men in El Salvador**. Aids and Behavior 2013, 17: 1279-1287.

65. King R, Barker J, Nakayiwa S, Katuntu D, Lubwama G, Bagenda D, Lane T, Opio A, Hladik W: **Men at Risk; a Qualitative Study on HIV Risk, Gender Identity and Violence among Men Who Have Sex with Men Who Report High Risk Behavior in Kampala, Uganda**. Plos One 2013, 8:

66. Manopaiboon C, Prybylski D, Subhachaturas W, Tanpradech S, Suksripanich O, Siangphoe U, Johnston LG, Akarasewi P, Anand A, Fox KK, Whitehead SJ: **Unexpectedly high HIV prevalence among female sex workers in Bangkok, Thailand in a respondent-driven sampling survey**. International Journal of Std & Aids 2013, 24: 34-38.

67. Murphy RD, Gorbach PM, Weiss RE, Hucks-Ortiz C, Shoptaw SJ: **Seroadaptation in a sample of very poor Los Angeles area men who have sex with men**. AIDS Behav 2013, 17: 1862-1872.

68. Mustanski B, Johnson AK, Garofalo R, Ryan D, Birkett M: **Perceived Likelihood of Using HIV Pre-exposure Prophylaxis Medications Among Young Men Who Have Sex with Men**. Aids and Behavior 2013, 17: 2173-2179.

69. Pando MA, Balan I, Marone R, Dolezal C, Barreda V, Dieguez AC, Avila MM: **HIV Knowledge and Beliefs Among Men Who Have Sex With Men (MSM) in Buenos Aires, Argentina**. Aids and Behavior 2013, 17: 1305-1312.

70. Park JN, Papworth E, Kassegne S, Moukam L, Billong SC, Macauley I, Yomb YR, Nkoume N, Mondoleba V, Eloundou J, LeBreton M, Tamoufe U, Grosso A, Baral SD: **HIV prevalence and factors associated with HIV infection among men who have sex with men in Cameroon**. J Int AIDS Soc 2013, 16 (Suppl 3): 18752

71. Risher K, Adams D, Sithole B, Ketende S, Kennedy C, Mnisi Z, Mabusa X, Baral SD: **Sexual stigma and discrimination as barriers to seeking appropriate healthcare among men who have sex with men in Swaziland**. J Int AIDS Soc 2013, 16: 18715

72. Rocha GM, Kerr LRFS, de Brito AM, Dourado I, Guimaraes MDC: **Unprotected Receptive Anal Intercourse Among Men Who have Sex with Men in Brazil**. Aids and Behavior 2013, 17: 1288-1295.

73. Wirtz AL, Jumbe V, Trapence G, Kamba D, Umar E, Ketende S, Berry M, Stromdahl S, Beyrer C, Baral SD: **HIV among men who have sex with men in Malawi: elucidating HIV prevalence and correlates of infection to inform HIV prevention**. J Int AIDS Soc 2013, 16 (Suppl 3): 18742

74. Yasin F, Delegchoimbol A, Jamiyanjamts N, Sovd T, Mason K, Baral S: **A Cross-Sectional Evaluation of Correlates of HIV Testing Practices Among Men Who Have Sex with Men (MSM) in Mongolia**. Aids and Behavior 2013, 17: 1378-1385.

75. Zhang L, Xiao Y, Lu RR, Wu GH, Ding XB, Qian HZ, McFarland W, Ruan YH, Vermund SH, Shao YM: **Predictors of HIV Testing Among Men Who Have Sex With Men in a Large Chinese City**. Sexually Transmitted Diseases 2013, 40: 235-240.

76. Zhou C, Raymond HF, Ding XB, Lu RR, Xu J, Wu GH, Feng LG, Fan S, Li XF, McFarland W, Xiao Y, Ruan YH, Shao YM: **Anal Sex Role, Circumcision Status, and HIV Infection Among Men Who Have Sex with Men in Chongqing, China**. Archives of Sexual Behavior 2013, 42: 1275-1283.

77. Zohrabyan L, Johnston LG, Scutelniciuc O, Iovita A, Todirascu L, Costin T, Plesca V, Cotelnic-Harea T, Ionascu G: **Determinants of HIV Infection Among Female Sex Workers in Two Cities in the Republic of Moldova: The Role of Injection Drug Use and Sexual Risk**. Aids and Behavior 2013, 17: 2588-2596.

78. Baral S, Burrell E, Scheibe A, Brown B, Beyrer C, Bekker LG: **HIV Risk and Associations of HIV Infection among men who have sex with men in Peri-Urban Cape Town, South Africa**. Bmc Public Health 2011, 11:

79. Bohl DD, Raymond HF, Arnold M, McFarland W: **Concurrent sexual partnerships and racial disparities in HIV infection among men who have sex with men**. Sexually Transmitted Infections 2009, 85: 367-369.

80. Burt RD,Thiede H: **Variations in Patterns of Sexual Risk Behavior among Seattle-Area MSM Based on their HIV Status, the HIV Status of their Partner and Partner Type**. Aids and Behavior 2012, 16: 599-607.

81. Chemnasiri T, Netwong T, Visarutratana S, Varangrat A, Li A, Phanuphak P, Jommaroeng R, Akarasewi P, van Griensven F: **Inconsistent Condom Use Among Young Men Who Have Sex with Men, Male Sex Workers, and Transgenders in Thailand**. Aids Education and Prevention 2010, 22: 100-109.

82. Chen YH, Vallabhaneni S, Raymond HF, McFarland W: **Predictors of Serosorting and Intention to Serosort Among Men Who Have Sex with Men, San Francisco**. Aids Education and Prevention 2012, 24: 564-573.

83. Choi KH, McFarland W, Neilands TB, Nguyen S, Secura G, Behel S, MacKellar D, Valleroy L: **High level of hepatitis B infection and ongoing risk among Asian/Pacific islander men who have sex with men, San Francisco, 2000-2001**. Sexually Transmitted Diseases 2005, 32: 44-48.

84. Do TD, Chen S, McDarland W, Secura GM, Behel SK, MacKellar DA, Valleroy LA, Choi KH: **HIV testing patterns and unrecognized HIV infection among young Asian and Pacific islander men who have sex with men in San Francisco**. Aids Education and Prevention 2005, 17: 540-554.

85. Forrest DW, Metsch LR, LaLota M, Cardenas G, Beck DW, Jeanty Y: **Crystal Methamphetamine Use and Sexual Risk Behaviors among HIV-Positive and HIV-Negative Men Who Have Sex with Men in South Florida**. Journal of Urban Health-Bulletin of the New York Academy of Medicine 2010, 87: 480-485.

86. Guadamuz TE, Wimonsate W, Varangrat A, Phanuphak P, Jommaroeng R, Mock PA, Tappero JW, van Griensven F: **Correlates of Forced Sex Among Populations of Men Who Have Sex with Men in Thailand**. Archives of Sexual Behavior 2011, 40: 259-266.

87. Holloway IW, Traube DE, Rice E, Schrager SM, Palinkas LA, Richardson J, Kipke MD: **Community and Individual Factors Associated With Cigarette Smoking Among Young Men Who Have Sex With Men**. Journal of Research on Adolescence 2012, 22: 199-205.

88. Jenness SM, Neaigus A, Murrill CS, Gelpi-Acosta C, Wendel T, Hagan H: **Recruitment-Adjusted Estimates of HIV Prevalence and Risk Among Men Who Have Sex with Men: Effects of Weighting Venue-Based Sampling Data**. Public Health Reports 2011, 126: 635-642.

89. Jenness SM, Neaigus A, Hagan H, Wendel T, Gelpi-Acosta C, Murrill CS: **Reconsidering the internet as an HIV/STD risk for men who have sex with men**. AIDS Behav 2010, 14: 1353-1361.

90. Kanter J, Koh C, Razali K, Tai R, Izenberg J, Rajan L, van Griensven F, Kamarulzaman A: **Risk behaviour and HIV prevalence among men who have sex with men in a multiethnic society: a venue-based study in Kuala Lumpur, Malaysia**. International Journal of Std & Aids 2011, 22: 30-37.

91. Kipke MD, Weiss G, Wong CF: **Residential status as a risk factor for drug use and HIV risk among young men who have sex with men**. Aids and Behavior 2007, 11: S56-S69.

92. Kipke MD, Kubicek K, Weiss G, Wong C, Lopez D, Iverson E, Ford W: **The health and health behaviors of young men who have sex with men**. Journal of Adolescent Health 2007, 40: 342-350.

93. Kipke MD, Weiss G, Ramirez M, Dorey F, Ritt-Olson A, Iverson E, Ford W: **Club drug use in Los Angeles among young men who have sex with men**. Substance Use & Misuse 2007, 42: 1723-1743.

94. Lim JR, Sullivan PS, Salazar L, Spaulding AC, DiNenno EA: **History of Arrest and Associated Factors among Men Who Have Sex with Men**. Journal of Urban Health-Bulletin of the New York Academy of Medicine 2011, 88: 677-689.

95. Lo YC, Turabelidze G, Lin M, Friedberg Y: **Prevalence and Determinants of Recent HIV Testing Among Sexually Active Men Who Have Sex With Men in the St. Louis Metropolitan Area, Missouri, 2008**. Sexually Transmitted Diseases 2012, 39: 306-311.

96. MacKellar DA, Gallagher KM, Finlayson T, Sanchez T, Lansky A, Sullivan PS: **Surveillance of HIV risk and prevention behaviors of men who have sex with men - A national application of venue-based, time-space sampling**. Public Health Reports 2007, 122: 39-47.

97. Mansergh G, Naorat S, Jommaroeng R, Jenkins RA, Stall R, Jeeyapant S, Phanuphak P, Tappero JW, van Griensven F: **Inconsistent condom use with steady and casual partners and associated factors among sexually-active men who have sex with men in Bangkok, Thailand**. Aids and Behavior 2006, 10: 743-751.

98. Mansergh G, Naorat S, Jommaroeng R, Jenkins RA, Jeeyapant S, Kanggarnrua K, Phanuphak P, Tappero JW, van Griensven F: **Adaptation of venue-day-time sampling in Southeast Asia to access men who have sex with men for HIV assessment in Bangkok**. Field Methods 2006, 18: 135-152.

99. McFarland W, Chen YH, Raymond HF, Binh N, Colfax G, Mehrtens J, Robertson T, Stall R, Levine D, Truong HHM: **HIV seroadaptation among individuals, within sexual dyads, and by sexual episodes, men who have sex with men, San Francisco, 2008**. Aids Care-Psychological and Socio-Medical Aspects of Aids/Hiv 2011, 23: 261-268.

100. Moore DM, Kanters S, Michelow W, Gustafson R, Hogg RS, Kwag M, Trussler T, McGuire M, Robert W, Gilbert M: **Implications for HIV Prevention Programs From a Serobehavioural Survey of Men Who Have Sex With Men in Vancouver, British Columbia: The ManCount Study**. Canadian Journal of Public Health-Revue Canadienne de Sante Publique 2012, 103: 142-146.

101. Neaigus A, Jenness SM, Hagan H, Murrill CS, Torian LV, Wendel T, Gelpi-Acosta C: **Estimating HIV Incidence and the Correlates of Recent Infection in Venue-Sampled Men Who Have Sex With Men in New York City**. Aids and Behavior 2012, 16: 516-524.

102. Newman PA, Chakrapani V, Cook C, Shunmugam M, Kakinami L: **Correlates of paid sex among men who have sex with men in Chennai, India**. Sexually Transmitted Infections 2008, 84: 434-438.

103. Newman PA, Chakrapani V, Cook C, Shunmugam M, Kakinami L: **Determinants of sexual risk behavior among men who have sex with men accessing public sex environments in Chennai, India**. J LGBT Health Res 2008, 4: 81-87.

104. Operario D, Choi KH, Chu PL, McFarland W, Secura GM, Behel S, MacKellar D, Valleroy L: **Prevalence and correlates of substance use among young Asian pacific islander men who have sex with men**. Prevention Science 2006, 7: 19-29.

105. Sheridan S, Phimphachanh C, Chanlivong N, Manivong S, Khamsyvolsvong S, Lattanavong P, Sisouk T, Toledo C, Scherzer M, Toole M, van Griensven F: **HIV prevalence and risk behaviour among men who have sex with men in Vientiane Capital, Lao People's Democratic Republic, 2007**. Aids 2009, 23: 409-414.

106. Sifakis F, Hylton JB, Flynn C, Solomon L, MacKellar DA, Valleroy LA, Celentano DD: **Prevalence of HIV Infection and Prior HIV Testing among Young Men Who have Sex with Men. The Baltimore Young Men's Survey**. Aids and Behavior 2010, 14: 904-912.

107. Snowden JM, Raymond HF, McFarland W: **Prevalence of seroadaptive behaviours of men who have sex with men, San Francisco, 2004**. Sexually Transmitted Infections 2009, 85: 469-476.

108. Toledo CA, Varangrat A, Wimolsate W, Chemnasiri T, Phanuphak P, Kalayil EJ, McNicholl J, Karuchit S, Kengkarnrua K, van Griensven F: **Examining Hiv Infection Among Male Sex Workers in Bangkok, Thailand: A Comparison of Participants Recruited at Entertainment and Street Venues**. Aids Education and Prevention 2010, 22: 299-311.

109. van Griensven F, Thanprasertsuk S, Jommaroeng R, Mansergh G, Naorat S, Jenkins RA, Ungchusak K, Phanuphak P, Tappero JW: **Evidence of a previously undocumented epidemic of HIV infection among men who have sex with men in Bangkok,Thailand**. Aids 2005, 19: 521-526.

110. van Griensven F,et al: **HIV prevalence among populations of men who have sex with men--Thailand, 2003 and 2005**. MMWR Morb Mortal Wkly Rep 2006, 55: 844-848.

111. Wei CY, Raymond HF, Guadamuz TE, Stall R, Colfax GN, Snowden JM, McFarland W: **Racial/Ethnic Differences in Seroadaptive and Serodisclosure Behaviors Among Men Who Have Sex with Men**. Aids and Behavior 2011, 15: 22-29.

112. Wong CF, Kipke MD, Weiss G: **Risk factors for alcohol use, frequent use, and binge drinking among young men who have sex with men**. Addictive Behaviors 2008, 33: 1012-1020.

113. Wong CF, Weiss G, Ayala G, Kipke MD: **Harassment, Discrimination, Violence, and Illicit Drug Use Among Young Men Who Have Sex with Men**. Aids Education and Prevention 2010, 22: 286-298.

114. Zhao J, Cai WD, Gan YX, Zhang Y, Yang ZR, Cheng JQ, Lin SH, He ML, Chen L, Wang XR: **A Comparison of HIV Infection and Related Risk Factors Between Money Boys and Noncommercial Men Who Have Sex With Men in Shenzhen, China**. Sexually Transmitted Diseases 2012, 39: 942-948.

115. Deiss RG, Clark JL, Konda KA, Leon SR, Klausner JD, Caceres CF, Coates TJ: **Problem drinking is associated with increased prevalence of sexual risk behaviors among men who have sex with men (MSM) in Lima, Peru**. Drug and Alcohol Dependence 2013, 132: 134-139.

116. Grov C, Ventuneac A, Rendina HJ, Jimenez RH, Parsons JT: **Recruiting Men Who Have Sex With Men on Craigslist.org for Face-to-Face Assessments: Implications for Research**. Aids and Behavior 2013, 17: 773-778.

117. Jung M: **Associations of Physical and Sexual Health with Suicide Attempts Among Female Sex Workers in South Korea**. Sexuality and Disability 2013, 31: 275-286.

118. Traube DE, Schrager SM, Holloway IW, Weiss G, Kipke MD: **Environmental risk, social cognition, and drug use among young men who have sex with men: Longitudinal effects of minority status on health processes and outcomes**. Drug and Alcohol Dependence 2013, 127: 1-7.

119. Wejnert C, Le B, Rose CE, Oster AM, Smith AJ, Zhu JL, The GPBF: **HIV Infection and Awareness among Men Who Have Sex with Men-20 Cities, United States, 2008 and 2011**. Plos One 2013, 8:

120. Balthasar H, Jeannin A, Dubois-Arber F: **First Anal Intercourse and Condom Use Among Men Who Have Sex with Men in Switzerland**. Archives of Sexual Behavior 2009, 38: 1000-1008.

121. Barron-Limon S, Semple SJ, Strathdee SA, Lozada R, Vargas-Ojeda A, Patterson TL: **Correlates of unprotected anal sex among men who have sex with men in Tijuana, Mexico**. Bmc Public Health 2012, 12:

122. Chen JL, Bovee MC, Kerndt PR: **Sexually transmitted diseases surveillance among incarcerated men who have sex with men - An opportunity for HIV prevention**. Aids Education and Prevention 2003, 15: 117-126.

123. Clark JL, Konda KA, Segura ER, Salvatierra HJ, Leon SR, Hall ER, Caceres CF, Klausner JD, Coates TJ: **Risk factors for the spread of HIV and other sexually transmitted infections among men who have sex with men infected with HIV in Lima, Peru**. Sexually Transmitted Infections 2008, 84: 449-454.

124. De SJ: **How do the sexual behaviors of foreign-born Hispanic men who have sex with men differ by relationship status?** Am J Mens Health 2012, 6: 6-17.

125. Du Bois SN, Emerson E, Mustanski B: **Condom-Related Problems Among a Racially Diverse Sample of Young Men Who Have Sex with Men**. Aids and Behavior 2011, 15: 1342-1346.

126. Elford J, Jeannin A, Spencer B, Gervasoni JP, van de Laar MJ, Dubois-Arber F: **HIV and STI behavioural surveillance among men who have sex with men in Europe**. Euro Surveill 2009, 14:

127. Flores SA, Mansergh G, Marks G, Guzman R, Colfax G: **Gay Identity Related Factors and Sexual Risk Among Men Who Have Sex with Men in San Francisco**. Aids Education and Prevention 2009, 21: 91-103.

128. Folch C, Munoz R, Zaragoza K, Casabona J: **Sexual risk behaviour and its determinants among men who have sex with men in Catalonia, Spain**. Euro Surveill 2009, 14:

129. Godin G, Bah AT, Sow A, Minani I, Morin D, Alary M: **Correlates of condom use among sex workers and their boyfriends in three West African countries**. Aids and Behavior 2008, 12: 441-451.

130. Guo HX, Wei JF, Yang HT, Huan XP, Tsui SKW, Zhang CY: **Rapidly Increasing Prevalence of HIV and Syphilis and HIV-1 Subtype Characterization Among Men Who Have Sex With Men in Jiangsu, China**. Sexually Transmitted Diseases 2009, 36: 120-125.

131. Halkitis PN, Moeller RW, Siconolfi DE, Jerome RC, Rogers M, Schillinger J: **Methamphetamine and poly-substance use among gym-attending men who have sex with men in New York City**. Ann Behav Med 2008, 35: 41-48.

132. He Q, Wang Y, Lin P, Liu YY, Yang F, Fu XB, Li Y, Sun BS, Li J, Zhao XX, Mandel J, Jain S, McFarland W: **Potential bridges for HIV infection to men who have sex with men in Guangzhou, China**. Aids and Behavior 2006, 10: S17-S23.

133. Hemmige V, Snyder H, Liao CH, Mayer K, Lakshmi V, Gandham SR, Orunganti G, Schneider J: **Sex Position, Marital Status, and HIV Risk Among Indian Men Who Have Sex with Men: Clues to Optimizing Prevention Approaches**. Aids Patient Care and Stds 2011, 25: 725-734.

134. Jarama SL, Kennamer JD, Poppen PJ, Hendricks M, Bradford J: **Psychosocial, behavioral, and cultural predictors of sexual risk for HIV infection among Latino men who have sex with men**. Aids and Behavior 2005, 9: 513-523.

135. Jimenez AD: **Triple jeopardy: Targeting older men of color who have sex with men**. Jaids-Journal of Acquired Immune Deficiency Syndromes 2003, 33: S222-S225.

136. Linhart Y, Shohat T, Amitai Z, Gefen D, Srugo I, Blumstein G, Dan M: **Sexually transmitted infections among brothel-based sex workers in Tel-Aviv area, Israel: high prevalence of pharyngeal gonorrhoea**. International Journal of Std & Aids 2008, 19: 656-659.

137. Mitchell JW,Petroll AE: **Patterns of HIV and Sexually Transmitted Infection Testing Among Men Who Have Sex With Men Couples in the United States**. Sexually Transmitted Diseases 2012, 39: 871-876.

138. Mor Z, Davidovich U, Bessudu-Manor N, McFarlane M, Feldshtein G, Chemtob D: **High-risk behaviour in steady and in casual relationships among men who have sex with men in Israel**. Sexually Transmitted Infections 2011, 87: 532-537.

139. Schneider JA, Michaels S, Gandham SR, McFadden R, Liao CH, Yeldandi VV, Oruganti G: **A Protective Effect of Circumcision Among Receptive Male Sex Partners of Indian Men Who Have Sex with Men**. Aids and Behavior 2012, 16: 350-359.

140. Seib C, Fischer J, Najman JM: **The health of female sex workers from three industry sectors in Queensland, Australia**. Social Science & Medicine 2009, 68: 473-478.

141. Setia MS, Lindan C, Jerajani HR, Kumta S, Ekstrand M, Mathur M, Gogate A, Kavi AR, Anand V, Klausner JD: **Men who have sex with men and transgenders in Mumbai, India: an emerging risk group for STIs and HIV**. Indian J Dermatol Venereol Leprol 2006, 72: 425-431.

142. Strathdee SA, Lozada R, Semple SJ, Crozovich P, Pu M, Staines-Orozco H, Fraga-Vallejo M, Amaro H, Delatorre A, Magis-Rodriguez C, Patterson TL: **Characteristics of female sex workers with US clients in two Mexico-US border cities**. Sexually Transmitted Diseases 2008, 35: 263-268.

143. Stromdahl S, Williams AO, Eziefule B, Emmanuel G, Iwuagwu S, Anene O, Orazulike I, Beyrer C, Baral S: **Associations of Consistent Condom Use Among Men Who Have Sex with Men in Abuja, Nigeria**. Aids Research and Human Retroviruses 2012, 28: 1756-1762.

144. Thornton AC, Lattimore S, Delpech V, Weiss HA, Elford J: **Circumcision Among Men Who Have Sex With Men in London, United Kingdom: An Unlikely Strategy for HIV Prevention**. Sexually Transmitted Diseases 2011, 38: 928-931.

145. Tinmouth J, Gilmour MW, Kovacs C, Kropp R, Mitterni L, Rachlis A, Richards S, Salit I, Sikri R, Valencia GR, Wesson T, Wong T, Wood H: **Is there a reservoir of sub-clinical lymphogranuloma venereum and non-LGV Chlamydia trachomatis infection in men who have sex with men?** Int J STD AIDS 2008, 19: 805-809.

146. Tripathi A, Ruutel K, Parker RD: **Hiv Risk Behaviour Knowledge, Substance Use and Unprotected Sex in Men Who Have Sex with Men in Tallinn, Estonia**. Eurosurveillance 2009, 14: 30-34.

147. Wang C, Wang YX, Huang XJ, Li X, Zhang T, Song MS, Wu LJ, Du J, Lu XQ, Shao S, Zhao FF, Ball MA, Wu H, Wang W: **Prevalence and Factors Associated with Hepatitis B Immunization and Infection among Men Who Have Sex with Men in Beijing, China**. Plos One 2012, 7:

148. Wilson EC, Garofalo R, Harris RD, Herrick A, Martinez M, Martinez J, Belzer M: **Transgender Female Youth and Sex Work: HIV Risk and a Comparison of Life Factors Related to Engagement in Sex Work**. Aids and Behavior 2009, 13: 902-913.

149. Yin YP, Chen SC, Wang HC, Wei WH, Wang QQ, Liang GJ, Jiang N, Han Y, Chen XS, Wang BX: **Prevalence and Risk Factors of HSV-2 Infection and HSV-2/HIV Coinfection in Men Who Have Sex With Men in China: A Multisite Cross-Sectional Study**. Sexually Transmitted Diseases 2012, 39: 354-358.

150. Zhou F, Gao L, Li SM, Li DL, Zhang LF, Fan WS, Yang XY, Yu MR, Xiao D, Yan L, Zhang Z, Shi W, Luo FJ, Ruan YH, Jin Q: **Willingness to Accept HIV Pre-Exposure Prophylaxis among Chinese Men Who Have Sex with Men**. Plos One 2012, 7:

151. Todd CS, Nasir A, Stanekzai MR, Scott PT, Close NC, Botros BA, Strathdee SA, Tjaden J: **HIV awareness and condom use among female sex workers in Afghanistan: implications for intervention**. Aids Care-Psychological and Socio-Medical Aspects of Aids/Hiv 2011, 23: 348-356.

152. Batist E, Brown B, Scheibe A, Baral SD, Bekker LG: **Outcomes of a community-based HIV-prevention pilot programme for township men who have sex with men in Cape Town, South Africa**. J Int AIDS Soc 2013, 16 (Suppl 3): 18754

153. Baral S, Adams D, Lebona J, Kaibe B, Letsie P, Tshehlo R, Wirtz A, Beyrer C: **A cross-sectional assessment of population demographics, HIV risks and human rights**

contexts among men who have sex with men in Lesotho. Journal of the International Aids Society 2011, 14:

154. Baral S, Kizub D, Masenior NF, Peryskina A, Stachowiak J, Stibich M, Moguilny V, Beyrer C: **Male sex workers in Moscow, Russia: a pilot study of demographics, substance use patterns, and prevalence of HIV-1 and sexually transmitted infections**. Aids Care-Psychological and Socio-Medical Aspects of Aids/Hiv 2010, 22: 112-118.

155. Baral S, Trapence G, Motimedi F, Umar E, Iipinge S, Dausab F, Beyrer C: **HIV Prevalence, Risks for HIV Infection, and Human Rights among Men Who Have Sex with Men (MSM) in Malawi, Namibia, and Botswana**. Plos One 2009, 4:

156. Bianchi FT, Shedlin MG, Brooks KD, Penha MM, Reisen CA, Zea MC, Poppen PJ: **Partner Selection among Latino Immigrant Men Who Have Sex with Men**. Archives of Sexual Behavior 2010, 39: 1321-1330.

157. Chersich MF, Luchters SMF, Malonza IM, Mwarogo P, King'ola N, Temmerman M: **Heavy episodic drinking among Kenyan female sex workers is associated with unsafe sex, sexual violence and sexually transmitted infections**. International Journal of Std & Aids 2007, 18: 764-769.

158. Choi KH, Hudes ES, Steward WT: **Social discrimination, concurrent sexual partnerships, and HIV risk among men who have sex with men in Shanghai, China**. Aids and Behavior 2008, 12: S71-S77.

159. Choi KH, Ning Z, Gregorich SE, Pan QC: **The influence of social and sexual networks in the spread of HIV and syphilis among men who have sex with men in Shanghai, China**. Jaids-Journal of Acquired Immune Deficiency Syndromes 2007, 45: 77-84.

160. de Souza CTV, Lowndes CM, Szwarcwald CL, Sutmoller F, Bastos FI: **Willingness to participate in HIV vaccine trials among a sample of men who have sex with men, with and without a history of commercial sex, Rio de Janeiro, Brazil**. Aids Care-Psychological and Socio-Medical Aspects of Aids/Hiv 2003, 15: 539-548.

161. Ding YP, Detels R, Zhao ZW, Zhu Y, Zhu GH, Zhang BW, Shen T, Xue XS: **HIV infection and sexually transmitted diseases in female commercial sex workers in China**. Jaids-Journal of Acquired Immune Deficiency Syndromes 2005, 38: 314-319.

162. Feng YJ, Wu ZY, Detels R, Qin GM, Liu L, Wang XD, Wang J, Zhang LL: **HIV/STD Prevalence Among Men Who Have Sex With Men in Chengdu, China and Associated Risk Factors for HIV Infection**. Jaids-Journal of Acquired Immune Deficiency Syndromes 2010, 53: S74-S80.

163. Garofalo R, Mustanski BS, McKirnan DJ, Herrick A, Donenberg GR: **Methamphetamine and young men who have sex with men - Understanding patterns and correlates of use and the association with HIV-related sexual risk**. Archives of Pediatrics & Adolescent Medicine 2007, 161: 591-596.

164. Goldsamt LA, Clatts MC, Parker MM, Colon V, Hallack R, Messina MG: **Prevalence of Sexually Acquired Antiretroviral Drug Resistance in a Community Sample of HIV-Positive Men Who Have Sex with Men in New York City**. Aids Patient Care and Stds 2011, 25: 287-293.

165. Gu J, Chen HY, Chen X, Lau JTF, Wang RF, Liu CL, Liu J, Lei ZQ, Li ZL: **Severity of drug dependence, economic pressure and HIV-related risk behaviors among non-institutionalized female injecting drug users who are also sex workers in China**. Drug and Alcohol Dependence 2008, 97: 257-267.

166. Gu J, Lau JTF, Chen HY, Tsui HY, Ling WH: **Prevalence and factors related to syringe sharing behaviours among female injecting drug users who are also sex workers in China**. International Journal of Drug Policy 2011, 22: 26-33.

167. Gu J, Lau JTF, Chen X, Liu CL, Liu J, Chen HY, Wang RF, Lei ZQ, Li ZL: **Using the Theory of Planned Behavior to investigate condom use behaviors among female injecting drug users who are also sex workers in China**. Aids Care-Psychological and Socio-Medical Aspects of Aids/Hiv 2009, 21: 967-975.

168. Han XX, Xu JJ, Chu ZX, Dai D, Lu CM, Wang X, Zhao L, Zhang C, Ji YT, Zhang H, Shang H: **Screening Acute HIV Infections among Chinese Men Who Have Sex with Men from Voluntary Counseling & Testing Centers**. Plos One 2011, 6:

169. Lau JTF, Yan HJ, Lin CQ, Zhang J, Choi KC, Wang ZJ, Hao C, Huan XP, Yang HT: **How Willing are Men Who Have Sex with Men in China to be Circumcised for the Sake of Protecting His Female Sex Partner?** Journal of Sexual Medicine 2012, 9: 1904-1912.

170. Lau JTF, Zhang J, Yan HJ, Lin CQ, Choi KC, Wang ZJ, Hao C, Huan XP, Yang HT: **Acceptability of circumcision as a means of HIV prevention among men who have sex with men in China**. Aids Care-Psychological and Socio-Medical Aspects of Aids/Hiv 2011, 23: 1472-1482.

171. Lorente N, Henry E, Fugon L, Yomb Y, Carrieri MP, Eboko F, Spire B: **Proximity to HIV is associated with a high rate of HIV testing among men who have sex with men living in Douala, Cameroon**. Aids Care-Psychological and Socio-Medical Aspects of Aids/Hiv 2012, 24: 1020-1027.

172. Lu F, Jia YJ, Sun XH, Wang L, Liu W, Xiao Y, Zeng G, Li CM, Liu JB, Cassell H, Chen HT, Vermund SH: **Prevalence of Hiv Infection and Predictors for Syphilis Infection Among Female Sex Workers in Southern China**. Southeast Asian Journal of Tropical Medicine and Public Health 2009, 40: 263-272.

173. Luchters S, Chersich MF, Rinyiru A, Barasa MS, King'ola N, Mandaliya K, Bosire W, Wambugu S, Mwarogo P, Temmerman M: **Impact of five years of peer-mediated interventions on sexual behavior and sexually transmitted infections among female sex workers in Mombasa, Kenya**. Bmc Public Health 2008, 8:

174. Luchters SMF, Vanden Broeck D, Chersich MF, Nel A, Delva W, Mandaliya K, Depuydt CE, Claeys P, Bogers JP, Temmerman M: **Association of HIV infection with distribution and viral load of HPV types in Kenya: a survey with 820 female sex workers**. Bmc Infectious Diseases 2010, 10:

175. Onyango-Ouma W, Birungi H, Geibel S: **Engaging men who have sex with men in operations research in Kenya**. Culture Health & Sexuality 2009, 11: 827-839.

176. Parry C, Petersen P, Dewing S, Carney T, Needle R, Kroeger K, Treger L: **Rapid assessment of drug-related HIV risk among men who have sex with men in three South African cities**. Drug and Alcohol Dependence 2008, 95: 45-53.

177. Peng B, Yang X, Zhang Y, Dai J, Liang H, Zou Y, Luo J, Peng H, Zhong X, Huang A: **Willingness to use pre-exposure prophylaxis for HIV prevention among female sex workers: a cross-sectional study in China**. HIV AIDS (Auckl) 2012, 4: 149-158.

178. Peterson JL, Rothenberg R, Kraft JM, Beeker C, Trotter R: **Perceived condom norms and HIV risks among social and sexual networks of young African American men who have sex with men**. Health Education Research 2009, 24: 119-127.

179. Ruan YH, Pan SW, Chamot E, Qian HZ, Li DL, Li QC, Liang HY, Spittal P, Shao YM, Kristensen A: **Sexual mixing patterns among social networks of HIV-positive and HIV-negative Beijing men who have sex with men: a multilevel comparison using roundtable network mapping**. Aids Care-Psychological and Socio-Medical Aspects of Aids/Hiv 2011, 23: 1014-1025.

180. Stulhofer A, Bacak V, Bozicevic I, Begovac J: **HIV-related sexual risk taking among HIV-negative men who have sex with men in Zagreb, Croatia**. Aids and Behavior 2008, 12: 505-512.

181. Thomas B, Mimiaga MJ, Mayer KH, Johnson CV, Menon S, Chandrasekaran V, Murugesan P, Swaminathan S, Safren SA: **HIV Prevention Interventions in Chennai, India: Are Men Who Have Sex with Men Being Reached?** Aids Patient Care and Stds 2009, 23: 981-986.

182. Todd CS, Alibayeva G, Khakimov MM, Sanchez JL, Bautista CT, Earhart KC: **Prevalence and correlates of condom use and HIV testing among female sex workers in Tashkent, Uzbekistan: Implications for HIV transmission**. Aids and Behavior 2007, 11: 435-442.

183. Trinks J, Cuestas ML, Tanaka Y, Mathet VL, Minassian ML, Rivero CW, Benetucci JA, Gimenez ED, Segura M, Bobillo MC, Corach D, Ghiringhelli PD, Sanchez DO, Avila MM, Peralta LAM, Kurbanov F, Weissenbacher MC, Simmonds P, Mizokami M, Oubina JR: **Two simultaneous hepatitis B virus epidemics among injecting drug users and men who have sex with men in Buenos Aires, Argentina: characterization of the first D/A recombinant from the American continent**. Journal of Viral Hepatitis 2008, 15: 827-838.

184. Zhang YQ, Chen PY, Lu RR, Liu L, Wu YZ, Liu XY, Zhao ZW, Yi D: **Prevalence of HIV among men who have sex with men in Chongqing, China, 2006-2009: cross-sectional biological and behavioural surveys**. Sexually Transmitted Infections 2012, 88: 444-450.

185. Ayoola OO, Sekoni AO, Odeyemi KA: **Transactional sex, condom and lubricant use among men who have sex with men in Lagos State, Nigeria**. Afr J Reprod Health 2013, 17: 90-98.

186. Chow EPF, Chen L, Jing J, Gao LM, Zhang J, Wilson DP, Zhang L: **HIV Disease Burden and Related Risk Behaviours Among Men Who Have Sex with Men in Yuxi Prefecture, Yunnan Province, China: 2010-2011**. Aids and Behavior 2013, 17: 2387-2394.

187. Mason K, Ketende S, Peitzmeier S, Ceesay N, Diouf D, Loum J, Deen D, Drame F, Baral S: **A Cross-Sectional Analysis of Population Demographics, HIV Knowledge and Risk Behaviors, and Prevalence and Associations of HIV Among Men Who Have Sex with Men in the Gambia**. Aids Research and Human Retroviruses 2013, 29: 1547-1552.

188. Song DD, Zhang HB, Wang J, Liu Q, Wang XD, Operario D, She M, Wang M, Zaller N: **Prevalence and Correlates of HIV Infection and Unrecognized HIV Status Among Men Who Have Sex with Men and Women in Chengdu and Guangzhou, China**. Aids and Behavior 2013, 17: 2395-2404.

189. Adam PCG, Teva I, De Wit JBF: **Balancing risk and pleasure: sexual self-control as a moderator of the influence of sexual desires on sexual risk-taking in men who have sex with men**. Sexually Transmitted Infections 2008, 84: 463-467.

190. Bauermeister JA, Carballo-Dieguez A, Ventuneac A, Dolezal C: **Assessing motivations to engage in intentional condomless anal intercourse in HIV risk contexts ("Bareback Sex") among men who have sex with men**. AIDS Educ Prev 2009, 21: 156-168.

191. Bowen A: **Internet sexuality research with rural men who have sex with men: can we recruit and retain them?** J Sex Res 2005, 42: 317-323.

192. Bull SS, Lloyd L, Rietmeijer C, McFarlane M: **Recruitment and retention of an online sample for an HIV prevention intervention targeting men who have sex with men: the Smart Sex Quest Project**. Aids Care-Psychological and Socio-Medical Aspects of Aids/Hiv 2004, 16: 931-943.

193. Chiasson MA, Shaw FS, Humberstone M, Hirshfield S, Hartel D: **Increased HIV disclosure three months after an online video intervention for men who have sex with men (MSM)**. Aids Care-Psychological and Socio-Medical Aspects of Aids/Hiv 2009, 21: 1081-1089.

194. Coleman E, Horvath KJ, Miner M, Ross MW, Oakes M, Rosser BRS: **Compulsive Sexual Behavior and Risk for Unsafe Sex Among Internet Using Men Who Have Sex with Men**. Archives of Sexual Behavior 2010, 39: 1045-1053.

195. Elford J, Doerner R, McKeown E, Nelson S, Anderson J, Low N: **HIV Infection Among Ethnic Minority and Migrant Men Who Have Sex With Men in Britain**. Sexually Transmitted Diseases 2012, 39: 678-686.

196. Fernandez MI, Perrino T, Collazo JB, Varga LM, Marsh D, Hernandez N, Rehbein A, Bowen GS: **Surfing new territory: club-drug use and risky sex among Hispanic men who have sex with men recruited on the Internet**. J Urban Health 2005, 82: i79-i88.

197. Hidaka Y, Ichikawa S, Koyano J, Urao M, Yasuo T, Kimura H, Ono-Kihara M, Kihara M: **Substance use and sexual behaviours of Japanese men who have sex with men: A nationwide internet survey conducted in Japan**. Bmc Public Health 2006, 6:

198. Hirshfield S, Chiasson MA, Wagmiller RL, Remien RH, Humberstone M, Scheinmann R, Grov C: **Sexual Dysfunction in an Internet Sample of US Men Who Have Sex with Men**. Journal of Sexual Medicine 2010, 7: 3104-3114.

199. Horvath KJ, Weinmeyer R, Rosser S: **Should it be illegal for HIV-positive persons to have unprotected sex without disclosure? An examination of attitudes among US men who have sex with men and the impact of state law**. AIDS Care 2010, 22: 1221-1228.

200. Jain A,Ross MW: **Predictors of Drop-Out in an Internet Study of Men Who Have Sex with Men**. Cyberpsychology & Behavior 2008, 11: 583-586.

201. Khosropour CM,Sullivan PS: **Predictors of Retention in an Online Follow-up Study of Men Who Have Sex With Men**. Journal of Medical Internet Research 2011, 13:

202. Krakower DS, Mimiaga MJ, Rosenberger JG, Novak DS, Mitty JA, White JM, Mayer KH: **Limited Awareness and Low Immediate Uptake of Pre-Exposure Prophylaxis among Men Who Have Sex with Men Using an Internet Social Networking Site**. Plos One 2012, 7:

203. Lim SH, Guadamuz TE, Wei CY, Chan R, Koe S: **Factors Associated with Unprotected Receptive Anal Intercourse with Internal Ejaculation Among Men Who Have Sex with Men in a Large Internet Sample from Asia**. Aids and Behavior 2012, 16: 1979-1987.

204. Marcus U, Schmidt AJ, Hamouda O, Bochow M: **Estimating the regional distribution of men who have sex with men (MSM) based on Internet surveys**. Bmc Public Health 2009, 9:

205. Mimiaga MJ, Reisner SL, Tinsley JP, Mayer KH, Safren SA: **Street Workers and Internet Escorts: Contextual and Psychosocial Factors Surrounding HIV Risk Behavior among Men Who Engage in Sex Work with Other Men**. Journal of Urban Health-Bulletin of the New York Academy of Medicine 2009, 86: 54-66.

206. Morgenstern J, Irwin TW, Wainberg ML, Parsons JT, Muench F, Bux DA, Kahler CW, Marcus S, Schulz-Heik J: **A randomized controlled trial of goal choice interventions for alcohol use disorders among men who have sex with men**. Journal of Consulting and Clinical Psychology 2007, 75: 72-84.

207. Mullens AB, Young RM, Dunne MP, Norton G: **The Amyl Nitrite Expectancy Questionnaire for Men who have Sex with Men (AEQ-MSM): A Measure of Substance-Related Beliefs**. Substance Use & Misuse 2011, 46: 1642-1650.

208. Mustanski B, Lyons T, Garcia SC: **Internet Use and Sexual Health of Young Men Who Have Sex with Men: A Mixed-Methods Study**. Archives of Sexual Behavior 2011, 40: 289-300.

209. Navejas M, Neaigus A, Torian L, Murrill C: **Participation in Online and Offline HIV Prevention Among Men who have Sex with Men who use the Internet to Meet Sex Partners in New York City**. Aids and Behavior 2012, 16: 389-395.

210. Ross MW, Mansson SA, Daneback K, Tikkanen R: **Characteristics of men who have sex with men on the Internet but identify as heterosexual, compared with heterosexually identified men who have sex with women**. Cyberpsychology & Behavior 2005, 8: 131-139.

211. Ross MW, Rosser BRS, Stanton J, Konstan J: **Characteristics of Latino men who have sex with men on the internet who complete and drop out of an Internet-based sexual behavior survey**. Aids Education and Prevention 2004, 16: 526-537.

212. Ruan YH, Jia YJ, Zhang XX, Liang HY, Li QC, Yang Y, Li DL, Zhou ZH, Luo FJ, Shi W, Shao YM: **Incidence of HIV-1, Syphilis, Hepatitis B, and Hepatitis C Virus Infections and Predictors Associated With Retention in a 12-Month Follow-Up Study Among Men Who Have Sex With Men in Beijing, China**. Jaids-Journal of Acquired Immune Deficiency Syndromes 2009, 52: 604-610.

213. Stephenson R, de VA, Sullivan PS: **Intimate Partner Violence and Sexual Risk-taking among Men Who Have Sex with Men in South Africa**. West J Emerg Med 2011, 12: 343-347.

214. Stephenson R, Khosropour C, Sullivan P: **Reporting of Intimate Partner Violence among Men Who Have Sex with Men in an Online Survey**. West J Emerg Med 2010, 11: 242-246.

215. Taylor BS, Chiasson MA, Scheinmann R, Hirshfield S, Humberstone M, Remien RH, Wolitski RJ, Wong T: **Results from Two Online Surveys Comparing Sexual Risk Behaviors in Hispanic, Black, and White Men Who Have Sex with Men**. Aids and Behavior 2012, 16: 644-652.

216. Tobin KE, German D, Spikes P, Patterson J, Latkin C: **A Comparison of the Social and Sexual Networks of Crack-Using and Non-Crack Using African American Men who Have Sex with Men**. Journal of Urban Health-Bulletin of the New York Academy of Medicine 2011, 88: 1052-1062.

217. Vet R, De Wit JBF, Das E: **The efficacy of social role models to increase motivation to obtain vaccination against hepatitis B among men who have sex with men**. Health Education Research 2011, 26: 192-200.

218. Zou HC, Wu ZY, Yu JP, Li M, Ablimit M, Li F, Pang L, Juniper N: **Sexual Risk Behaviors and HIV Infection Among Men Who Have Sex With Men Who Use the Internet in Beijing and Urumqi, China**. Jaids-Journal of Acquired Immune Deficiency Syndromes 2010, 53: S81-S87.

219. Berg RC, Ross MW, Weatherburn P, Schmidt AJ: **Structural and environmental factors are associated with internalised homonegativity in men who have sex with men: Findings from the European MSM Internet Survey (EMIS) in 38 countries**. Social Science & Medicine 2013, 78: 61-69.

220. Christensen JL, Miller LC, Appleby PR, Corsbie-Massay C, Godoy CG, Marsella SC, Read SJ: **Reducing shame in a game that predicts HIV risk reduction for young adult MSM: a randomized trial delivered nationally over the Web**. J Int AIDS Soc 2013, 16: 18716

221. Tobin KE, Latkin CA, Curriero FC: **An examination of places where African American men who have sex with men (MSM) use drugs/drink alcohol: A focus on social and spatial characteristics**. Int J Drug Policy 2013,

222. Baars JE, Boon BJ, Garretsen HF, van de Mheen D: **The reach of a hepatitis B vaccination programme among men who have sex with men**. European Journal of Public Health 2011, 21: 333-337.

223. Clatts MC, Goldsamt LA, Yi H: **Club drug use among young men who have sex with men in NYC: A preliminary epidemiological profile**. Substance Use & Misuse 2005, 40: 1317-1330.

224. Gwadz MV, Clatts MC, Leonard NR, Goldsamt L: **Attachment style, childhood adversity, and behavioral risk among young men who have sex with men**. Journal of Adolescent Health 2004, 34: 402-413.

225. Gwadz MV, Clatts MC, Yi H, Leonard NR, Goldsamt L, Lankenau S: **Resilience Among Young Men Who Have Sex With Men in New York City**. Sex Res Social Policy 2006, 3: 13-21.

226. Halkitis PN, Parsons JT, Wolitski RJ, Remien RH: **Characteristics of HIV antiretroviral treatments, access and adherence in an ethnically diverse sample of men who have sex with men**. Aids Care-Psychological and Socio-Medical Aspects of Aids/Hiv 2003, 15: 89-102.

227. Inciardi JA, Surratt HL, Kurtz SP: **HIV, HBV, and HCV infections among drug-involved, inner-city, street sex workers in Miami, Florida**. Aids and Behavior 2006, 10: 139-147.

228. Irwin TW,Morgenstern J: **Drug-use patterns among men who have sex with men presenting for alcohol treatment: Differences in ethnic and sexual identity**. Journal of Urban Health-Bulletin of the New York Academy of Medicine 2005, 82: I127-I133.

229. Landovitz RJ, Fletcher JB, Inzhakova G, Lake JE, Shoptaw S, Reback CJ: **A Novel Combination HIV Prevention Strategy: Post-Exposure Prophylaxis with Contingency Management for Substance Abuse Treatment Among Methamphetamine-Using Men Who Have Sex with Men**. Aids Patient Care and Stds 2012, 26: 320-328.

230. Pollock JA, Halkitis PN, Moeller RW, Solomon TM, Barton SC, Blachman-Forshay J, Siconolfi DE, Love HT: **Alcohol Use Among Young Men Who Have Sex With Men**. Substance Use & Misuse 2012, 47: 12-21.

231. Surratt HL, Inciardi JA, Kurtz SP, Kiley MC: **Sex work and drug use in a subculture of violence**. Crime & Delinquency 2004, 50: 43-59.

232. Surratt HL,Inciardi JA: **HIV risk, seropositivity and predictors of infection among homeless and non-homeless women sex workers in Miami, Florida, USA**. Aids Care-Psychological and Socio-Medical Aspects of Aids/Hiv 2004, 16: 594-604.

233. Surratt HL, Inciardi JA, Kurtz SP: **Prescription opioid abuse among drug-involved street-based sex workers**. J Opioid Manag 2006, 2: 283-289.

234. Surratt HL,Inciardi JA: **An effective HIV risk-reduction protocol for drug-using female sex workers**. J Prev Interv Community 2010, 38: 118-131.

235. Wechsberg WM, Luseno WK, Lam WK: **Violence against substance-abusing South African sex workers: intersection with culture and HIV risk**. Aids Care-Psychological and Socio-Medical Aspects of Aids/Hiv 2005, 17: S55-S64.

236. Muessig KE, Pike EC, Fowler B, LeGrand S, Parsons JT, Bull SS, Wilson PA, Wohl DA, Hightow-Weidman LB: **Putting prevention in their pockets: developing mobile phone-based HIV interventions for black men who have sex with men**. AIDS Patient Care STDS 2013, 27: 211-222.

237. Allman D, Adebajo S, Myers T, Odumuye O, Ogunsola S: **Challenges for the sexual health and social acceptance of men who have sex with men in Nigeria**. Culture Health & Sexuality 2007, 9: 153-168.

238. Boyce S, Barrington C, Bolanos H, Arandi CG, Paz-Bailey G: **Facilitating access to sexual health services for men who have sex with men and male-to-female transgender persons in Guatemala City**. Culture Health & Sexuality 2012, 14: 313-327.

239. Ghose T, Swendeman D, George S, Chowdhury D: **Mobilizing collective identity to reduce HIV risk among sex workers in Sonagachi, India: The boundaries, consciousness, negotiation framework**. Social Science & Medicine 2008, 67: 311-320.

240. Rhodes SD, Hergenrather KC, Aronson RE, Bloom FR, Felizzola J, Wolfson M, Vissman AT, Alonzo J, Allen AB, Montano J, McGuire J: **Latino men who have sex with men and HIV in the rural south-eastern USA: findings from ethnographic in-depth interviews**. Culture Health & Sexuality 2010, 12: 797-812.

241. Wu J, Zhou XL, Lu CY, Moyer E, Wang H, Hong LY, Deng XQ: **A Qualitative Exploration of Barriers to Condom Use among Female Sex Workers in China**. Plos One 2012, 7:

242. Bengtsson L, Thorson A, Thanh VPN, Allebeck P, Popenoe R: **Sexual relationships among men who have sex with men in Hanoi, Vietnam: a qualitative interview study**. Bmc Public Health 2013, 13:

243. Fendrich M, Mackesy-Amiti ME, Johnson TP: **Validity of Self-Reported Substance Use in Men Who Have Sex With Men: Comparisons With a General Population Sample**. Annals of Epidemiology 2008, 18: 752-759.

244. Mackesy-Amiti ME, Fendrich M, Johnson TP: **Substance-related problems and treatment among men who have sex with men in comparison to other men in Chicago**. Journal of Substance Abuse Treatment 2009, 36: 227-233.

245. Tucker JS, Hu JH, Golinelli D, Kennedy DP, Green HD, Wenzel SL: **Social Network and Individual Correlates of Sexual Risk Behavior Among Homeless Young Men Who Have Sex With Men**. Journal of Adolescent Health 2012, 51: 386-392.

246. Webster RD, Darrow WW, Paul JP, Roark RA, Woods WJ, Stempel RR: **HIV infection and associated risks among young men who have sex with men in a Florida resort community**. Jaids-Journal of Acquired Immune Deficiency Syndromes 2003, 33: 223-231.

247. Cai Y, Shi R, Shen TA, Pei B, Jiang XQ, Ye XX, Xu G, Li SH, Huang H, Shang ML: **A study of HIV/AIDS related knowledge, attitude and behaviors among female sex workers in Shanghai China**. Bmc Public Health 2010, 10:

248. Jung M: **Sexual, Behavioral, and Social Characteristics of Female Sex Workers and Their Risk of Sexually Transmitted Infections: In South Korea**. Sexuality and Disability 2012, 30: 421-431.

249. Ye XX, Shang ML, Shen T, Pei B, Jiang XQ, Cai Y: **Social, psychological, and environmental-structural factors determine consistent condom use among rural-to-urban migrant female sex workers in Shanghai China**. Bmc Public Health 2012, 12:

250. Parsons JT, Kutnick AH, Halkitis PN, Punzalan JC, Carbonari JP: **Sexual risk behaviors and substance use among alcohol abusing HIV-positive men who have sex with men**. Journal of Psychoactive Drugs 2005, 37: 27-36.

251. Stulhofer A, Lausevic D, Bozicevic I, Bacak V, Mugosa B, Terzic N, Drglin T: **HIV Risks among Female Sex Workers in Croatia and Montenegro**. Collegium Antropologicum 2010, 34: 881-886.

252. Wong WCW, Leung PWS, Li CW: **HIV behavioural risks and the role of work environment among Chinese male sex workers in Hong Kong**. Aids Care-Psychological and Socio-Medical Aspects of Aids/Hiv 2012, 24: 340-347.

253. Fernandez MI, Warren JC, Varga LM, Prado G, Hernandez N, Bowen GS: **Cruising in cyber space: comparing Internet chat room versus community venues for recruiting Hispanic men who have sex with men to participate in prevention studies**. J Ethn Subst Abuse 2007, 6: 143-162.

254. Grov C: **HIV Risk and Substance Use in Men Who Have Sex with Men Surveyed in Bathhouses, Bars/Clubs, and on Craigslist.org: Venue of Recruitment Matters**. Aids and Behavior 2012, 16: 807-817.

255. Carrico AW, Pollack LM, Stall RD, Shade SB, Neilands TB, Rice TM, Woods WJ, Moskowitz JT: **Psychological processes and stimulant use among men who have sex with men**. Drug and Alcohol Dependence 2012, 123: 79-83.

256. Greenwood GL, Paul JP, Pollack LM, Binson D, Catania JA, Chang J, Humfleet G, Stall R: **Tobacco use and cessation among a household-based sample of US urban men who have sex with men**. American Journal of Public Health 2005, 95: 145-151.

257. Pisani E, Girault P, Gultom M, Sukartini N, Kumalawati J, Jazan S, Donegan E: **HIV, syphilis infection, and sexual practices among transgenders, male sex workers, and other men who have sex with men in Jakarta, Indonesia**. Sexually Transmitted Infections 2004, 80: 536-540.

258. Doerner R, McKeown E, Nelson S, Anderson J, Low N, Elford J: **Circumcision and HIV Infection among Men Who Have Sex with Men in Britain: The Insertive Sexual Role**. Archives of Sexual Behavior 2013, 42: 1319-1326.

259. Schwarcz S, Spindler H, Scheer S, Valleroy L, Lansky A: **Assessing representativeness of sampling methods for reaching men who have sex with men: A direct comparison of results obtained from convenience and probability samples**. Aids and Behavior 2007, 11: 596-602.

260. He Q, Wang Y, Li Y, Zhang YR, Lin P, Yang F, Fu XB, Li J, Raymond HF, Ling L, McFarland W: **Accessing men who have sex with men through long-chain referral recruitment, Guangzhou, China**. Aids and Behavior 2008, 12: S93-S96.

261. Xiao Y, Ding X, Li C, Liu J, Sun J, Jia Y: **Prevalence and correlates of HIV and syphilis infections among men who have sex with men in Chongqing Municipality, China**. Sex Transm Dis 2009, 36: 647-656.

262. Weir SS, Merli MG, Li J, Gandhi AD, Neely WW, Edwards JK, Suchindran CM, Henderson GE, Chen XS: **A comparison of respondent-driven and venue-based sampling of female sex workers in Liuzhou, China**. Sexually Transmitted Infections 2012, 88: I95-I101.

263. Paz-Bailey G, Miller W, Shiraishi RW, Jacobson JO, Abimbola TO, Chen SY: **Reaching Men Who Have Sex with Men: A Comparison of Respondent-Driven Sampling and Time-Location Sampling in Guatemala City**. Aids and Behavior 2013, 17: 3081-3090.

264. Evans AR, Wiggins RD, Mercer CH, Bolding GJ, Elford J: **Men who have sex with men in Great Britain: comparison of a self-selected internet sample with a national probability sample**. Sexually Transmitted Infections 2007, 83: 200-205.

265. Parsons JT, Vicioso KJ, Punzalan JC, Halkitis PN, Kutnick A, Velasquez MM: **The impact of alcohol use on the sexual scripts of HIV-positive men who have sex with men**. Journal of Sex Research 2004, 41: 160-172.

266. Mimiaga MJ, Reisner SL, Bland SE, Driscoll MA, Cranston K, Isenberg D, Vanderwarker R, Mayer KH: **Sex Parties among Urban MSM: An Emerging Culture and HIV Risk Environment**. Aids and Behavior 2011, 15: 305-318.

267. Gondim RC, Kerr LRFS, Werneck GL, Macena RHM, Pontes MK, Kendall C: **Risky sexual practices among men who have sex with men in Northeast Brazil: results from four sequential surveys**. Cadernos de Saude Publica 2009, 25: 1390-1398.

268. Chua AC, Chen MIC, Cavailler P, Jiang LL, Abdullah MR, Ng OT, Chio M, Koe S, Tay J, Wong ML, Chan R: **Challenges of Respondent Driven Sampling to Assess Sexual Behaviour and Estimate the Prevalence of Human Immunodeficiency Virus (HIV) and Syphilis in Men Who Have Sex with Men (MSM) in Singapore**. Annals Academy of Medicine Singapore 2013, 42: 350-353.
