# Supplementary material for: Hard-to-reach populations of men who have sex with men and sex workers: a systematic review on sampling methods
Source: Syst Rev. 2015 Oct 30;4:141. doi: 10.1186/s13643-015-0129-9 (PMC4627393; doi:10.1186/s13643-015-0129-9)
Supplement: Additional file 5: Text S4. — Sampling methods identified in the systematic literature review. [file 13643_2015_129_MOESM5_ESM.pdf]

Text S4: Sampling methods identified in the systematic review

Convenience sampling method is set of techniques in which respondents are selected by convenience due to their proximity, availability, accessibility or other way that researcher decides [1]. It is a fast and easy method to use however results seldom are representative of the population [2].

Purposive sampling is a technique in which researcher, with a particular purpose, chooses the respondents that participate in the study [1]. It is useful when researchers are looking for persons with a specific characteristic (sometimes rare) but, because it is a non-probability based sampling it does not allow to make inferences to the population.

Snowball sampling is a method of chain referral in which researchers contact members of the target population, already known to them, who are invited to participate in the survey and are in turn asked to refer their peers and/or help researchers to identify them [3]. The chain referral method allows researchers reach populations that are hard to sample using other methods, nevertheless individuals who have a large number of social connections are able to nominate peers that have the same characteristics as their own, and thus the sample may not be representative of the population[4].

Targeted sampling consists in doing an initial ethnographic assessment in order to identify subpopulations or subgroups with some specific attributes and then elaborate a plan to recruit members with those characteristics where the members of the population might be found [5]. This method offers an approach that can be useful to identify social and cultural characteristics of subgroups as well as their geographic distribution, but the sample will be biased towards those who gather in the selected location [5], is labour intensive and time consuming.

Random Digit Dialling (RDD) is a probabilistic method used for selecting respondents from a set of telephone numbers (usually landlines) [6]. It has the advantage of reaching a geographic dispersed sample and including those who live in rural areas, it is time and cost saving because interviewers do not have to physically go to study

areas. Among limitations of RDD there is the possible lack of representativeness, as not all persons have a landline, and the high rate of unfruitful calls [7].

Multi-stage sampling is a probabilistic method that consists of more than two stages of sampling; the first stage identifies the primary sampling units (PSUs) (e.g. geographical areas), the second stage selects the units within the PSU (Second Sampling Units – SSUs)(e.g. hospitals within geographical areas), the third stage selects the units within the SSUs (e.g. hospital units), and so on [8].

Cluster sampling is a special case of a multi-stage sampling, where the study population is divided into groups (or clusters) and then a sample of those clusters is selected[8,9].

In stratified probability sampling method the population members are divided into homogeneous groups (called strata) such as regions or age categories for instance, and then a sample is selected (usually a simple random sampling or systematic sampling) within each strata[9].

When specifically talking about surveying HRP, all probabilistic methods, like those presented here have the great disadvantage of the cost because most hidden populations, like MSM and FSW, are a minority in the general population, thus collecting a probability sample would be too expensive [2].

Internet sampling technique consists in recruiting respondents through the internet, either through advertising or contact people directly (through chat rooms for instance); we categorized in this method all publications that mentioned that respondents were recruited through “internet”, “on-line” or via “web”, no matter if it was a web-based survey or if the internet was used only to advertise the research [10]. This method offers a mechanism through which a researcher can have access to people who share similar characteristics, interests or attitudes. It is time and cost saving however little may be known about the characteristics of the online community members; it may not be easy to define a sampling frame if the researcher does not have access to the number of members of a community or their email addresses. Non-response rates are also hard to identify for most online communities [11].

Time Location Sampling (TLS) also called Time Space Sampling (TSS) consists in identifying the venues and time periods where the study population congregates and then select a sample of sites to recruit members during a pre-defined time interval[12]. TLS is an efficient way to sample hidden populations that congregate in specific locations and is able to approximate probability sampling [13], however not all select places are easily accessible and others are not even contemplated due to safety reasons or to high costs [14]. Besides, populations that congregate at public venues may differ from the true population as some of them may only frequent private venues. This means that there might be an unknown potential bias in the estimates [14].

Respondent Driven Sampling (RDS) is chain referral technique in which researchers contact a predefined small number of population members called seeds and asks them to recruit their peers to participate in the survey. If the peers are eligible for the study they are invited to become seeds and to recruit other members. This technique gives incentives to peers and to seeds who recruits them [15]. RDS has the advantage of recruiting individuals that do not congregate in public venues, however it will not function if the study population are not socially networked [16]. A major difference from Snowball sampling is that RDS relies on elements of the target population to recruit their peers using a set of coded coupons which are redeemed. The coupon quota reduces biases associated with over representation of those who have large networks [4]. Also in order to reduce bias, during the survey, data on network structure are also collected and used to determine post-hoc sampling weights[17].

References

1. Abrams LS: **Sampling 'Hard to Reach' Populations in Qualitative Research: The Case of Incarcerated Youth**. Qualitative Social Work 2010, 536

2. Meyer IH,Wilson PA: **Sampling Lesbian, Gay, and Bisexual Populations**. Journal of Counseling Psychology 2009, 56: 23-31.

3. Faugier J,Sargeant M: **Sampling hard to reach populations**. J Adv Nurs 1997, 26: 790-797.

4. Johnston L,Sabin K: **Sampling hard-to-reach populations with respondent driven sampling** . Methodological Innovations Online 2010, 5(2): 38-48.

5. Watters JK,Biernacki P: **Targeted Sampling - Options for the Study of Hidden Populations**. Social Problems 1989, 36: 416-430.

6. Link MW, Battaglia MP, Frankel MR, Osborn L, Mokdad AH: **A comparison of address-based sampling (ABS) versus random-digit dialing (RDD) for general population surveys**. Public Opinion Quarterly 2008, 72: 6-27.

7. Felix-Medina MH,Monjardin PE: **Combining Link-Tracing Sampling and Cluster Sampling to Estimate Totals and Means of Hidden Human Populations**. Journal of Official Statistics 2010, 26: 603-631.

8. Sarndal C-E, Swensson B,Wretman J: **Model Assisted Survey Sampling.** Springer; 1992.

9. Cochran WG: **Sampling Techniques.** 1977.

10. Raymond HF, Rebchook G, Curotto A, Vaudrey J, Amsden M, Levine D, McFarland W: **Comparing internet-based and venue-based methods to sample MSM in the San Francisco Bay Area**. AIDS Behav 2010, 14: 218-224.

11. Wright KB: **Researching Internet-based populations: Advantages and disadvantages of online survey research, online questionnaire authoring software packages, and Web survey services**. Journal of Computer-Mediated Communication 2005, 10:

12. Magnani R, Sabin K, Saidel T, Heckathorn D: **Review of sampling hard-to-reach and hidden populations for HIV surveillance**. Aids 2005, 19: S67-S72.

13. Kendall C, Kerr LR, Gondim RC, Werneck GL, Macena RH, Pontes MK, Johnston LG, Sabin K, McFarland W: **An empirical comparison of respondent-driven sampling, time location sampling, and snowball sampling for behavioral surveillance in men who have sex with men, Fortaleza, Brazil**. AIDS Behav 2008, 12: S97-104.

14. Salganik MJ,Heckathorn DD: **Sampling and estimation in hidden populations using respondent-driven sampling**. Sociological Methodology, 2004, Vol 34 2004, 34: 193-239.

15. Heckathorn DD: **Respondent-driven sampling: A new approach to the study of hidden populations**. Social Problems 1997, 44: 174-199.

16. Johnston LG, Malekinejad M, Kendall C, Iuppa IM, Rutherford GW: **Implementation challenges to using respondent-driven sampling methodology for HIV biological and behavioral surveillance: field experiences in international settings**. AIDS Behav 2008, 12: S131-S141.

17. Heckathorn DD: **Respondent-driven sampling II: Deriving valid population estimates from chain-referral samples of hidden populations**. Social Problems 2002, 49: 11-34.
